# Shape Memory Polymer Bioglass Composite Scaffolds Designed to Heal Complex Bone Defects

**DOI:** 10.1021/acsbiomaterials.4c01073

**Published:** 2024-10-04

**Authors:** Brandon M. Nitschke, Elizabeth A. Butchko, MaryGrace N. Wahby, Kaylee M. Breining, Alexander E. Konz, Melissa A. Grunlan

**Affiliations:** † Department of Biomedical Engineering, Texas A&M University, College Station, Texas 77843, United States; ‡ Department of Materials Science and Engineering, Texas A&M University, College Station, Texas 77843, United States; § Department of Chemistry, Texas A&M University, College Station, Texas 77843, United States

**Keywords:** bioglass, scaffold, bioceramic, composite, bone regeneration, bioactivity, polymer

## Abstract

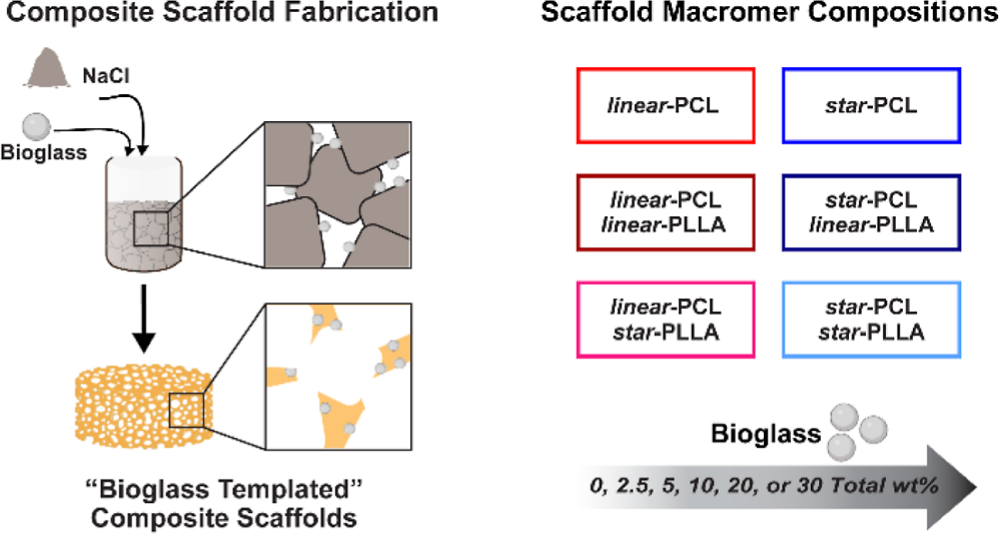

An off-the-shelf scaffold with requisite properties could enable the viable treatment of irregular craniomaxillofacial bone defects. Notably, the scaffold should be conformally fitting, innately bioactive, and bioresorbable. In prior work, we developed a series of shape memory polymer (SMP) scaffolds based on cross-linked poly(ε-caprolactone) (PCL). These were capable of “self-fitting” into complex bone defects when exposed to temperatures above the melt transition of the constituent PCL, either *linear*-PCL-diacrylate (*linear*-PCL-DA, *T*_m_ ∼55 °C) or *star*-PCL-tetraacrylate (*star*-PCL-TA, *T*_m_ ∼45 °C) for the potential to improve tissue safety. To achieve favorably increased degradation rates versus PCL-only scaffolds, semi-interpenetrating networks (semi-IPNs) were formed by including *linear*- or *star*-poly(l-lactic acid) (PLLA). A potential limitation of these self-fitting scaffolds is the lack of bioactivity, which is essential to osteoinductivity and osseointegration. Herein, analogous composite scaffolds were formed with 45S5 bioglass (BG) to impart bioactivity. The solvent-cast particulate leaching fabrication method was adapted to introduce BG to the fused salt template, resulting in composites with BG concentrated on the pore wall surfaces rather than within pore struts. Composite scaffolds with good pore wall integrity were produced with 2.5, 5, and 10 wt % BG. All composite scaffolds exhibited non-brittle behavior and did not fracture with 85% strain. For semi-IPN composite scaffolds, PLLA crystallinity was lost, and mechanical properties were not appreciably altered versus the non-BG controls. Sufficient retention of PCL crystallinity led to excellent shape memory behavior. The inclusion of 5 and 10 wt % BG led to hydroxyapatite mineralization after 1 day of exposure to simulated body fluid, as well as increased rates of in vitro degradation.

## 1 Introduction

Treatment of irregular craniomaxillofacial (CMF) bone defects with biological grafts is challenging due to limited conformal fitting (i.e., insufficient bone-to-graft contact) that leads to premature graft resorption.^1−8^ A regenerative engineering strategy that utilizes an off-the-shelf scaffold capable of conformally fitting is an attractive alternative. The resulting good bone-to-scaffold contact would be expected to promote osseointegration with adjacent tissue. Furthermore, a scaffold that is also bioactive would enhance regeneration via the formation of carbonated hydroxyapatite (HAp) [Ca_10_(PO_4_)_6_(OH)_2_],^9−11^ which is known to promote mesenchymal stem cell (MSC) differentiation into osteogenic cells (i.e., osteoinductivity).^12^ Bioactive scaffolds are typically prepared as composites, wherein a bioactive bioceramic is combined within a polymer matrix to reduce brittleness and postsurgical fracture of bioceramic-only systems.^12−14^ Such composite scaffolds may also achieve accelerated degradation rates that better align with neotissue formation, thereby enhancing osteoconductivity.^15,16^ While numerous bioactive bioceramics (e.g., tricalcium phosphate, HAp) have been evaluated, 45S5 bioglass (BG) [SiO_2_–Na_2_O–CaO–P_2_O_5_] has been widely studied since it was first reported decades ago.^11,17^ BG is associated with high bioactive potency, which allows for a HAp layer to form on the surface in a matter of hours via the exchange of ions between itself and physiological solutions.^11,12,18^ Overall, a bioactive composite scaffold capable of conformal fitting, robust HAp mineralization, and enhanced rates of degradation would represent a significant advancement in the treatment of irregular bone defects.

We have reported on “self-fitting” scaffolds for a regenerative strategy to heal irregular CMF bone defects.^19−21^ These thermoresponsive, shape memory polymer (SMP) scaffolds were prepared from biodegradable poly(ε-caprolactone) (PCL). Scaffolds were fabricated from UV-curable PCL macromers via a solvent-cast particulate leaching (SCPL) technique, leading to high porosity (∼70%) and interconnected macropores (*d* ∼ 220 μm). The crystalline lamellae of PCL serve as switching segments while the cross-links act as net-points. When exposed to saline above its *T*_m_, the scaffold becomes malleable and can be press-fit into an irregular defect. A conformal fit is achieved via shape recovery (i.e., expansion) of the scaffold that drives it outward to the defect’s perimeter. Once cooled below its *T*_m_, the scaffold returns to a rigid state and is locked into place (i.e., shape fixity).^19^ Furthermore, the *T*_m_ of PCL (i.e., fitting temperature) can be tuned depending on the architecture and *M*_n_ of the macromer.^22,23^ Scaffolds based on 2-arm *linear*-PCL-diacrylate (*linear*-PCL-DA, *M*_n_ ∼ 10 kg mol^–1^) have a *T*_m_ of ∼55 °C, while those prepared from 4-arm *star*-PCL-tetraacrylate (*star*-PCL-TA, *M*_n_ ∼ 10 kg mol^–1^) have a *T*_m_ of ∼45 °C. This lower *T*_m_ of *star*-PCL-TA is expected to limit potential necrosis to surrounding tissue during implantations that require prolonged irrigation to extend working time.^24^ Additionally, SMP scaffolds have been formed as semi-interpenetrating networks (semi-IPNs) by incorporating 25 wt % of thermoplastic (i.e., un-cross-linked) poly(l-lactic acid) [*linear*-poly(l-lactic acid) (PLLA) or *star*-PLLA, *M*_n_ ∼ 15 kg mol^–1^].^22^ These semi-IPN scaffolds exhibited increased degradation rates and, in some instances, greater compressive moduli.

While achieving numerous favorable properties, the previously reported PCL-based SMP scaffolds lack innate bioactivity that may limit their bone healing efficacy. Several strategies have thus been previously explored to produce bioactive SMP scaffolds. First, a polydopamine (PDA) coating was applied to the scaffold surface, and HAp was shown to have subsequently formed after 2 weeks in 1× simulated body fluid (SBF).^19,25^ However, a PDA-coating (*t* ∼ 50 nm) is lost as the scaffold degrades, thereby diminishing bioactivity over time. To impart bioactivity to the scaffold bulk, silicon-containing, UV-curable co-macromers based on polydimethylsiloxane (PDMS)^26^ and polymethylhydrosiloxane (PMHS)^27^ have also been incorporated to form co-networks with PCL. Mineralization (1× SBF) was also observed after 4 weeks for PCL–PDMS, and just 2 weeks for PCL–PMHS scaffolds. However, the low glass transition temperatures (*T*_g_s) of such macromers (*T*_g,PDMS_ = ∼−120 °C; *T*_g,PMHS_ = ∼−135 °C) led to a reduction in the premineralized scaffold modulus. Despite the scaffold bioactivity observed, HAp mineralization was comparatively slow (i.e., 2–4 weeks) versus that of bioactive fillers such as BG (i.e., hours to days).^12^ Thus, bioactive SMP scaffolds may be more effectively produced with the inclusion of BG.

**Figure 1:**
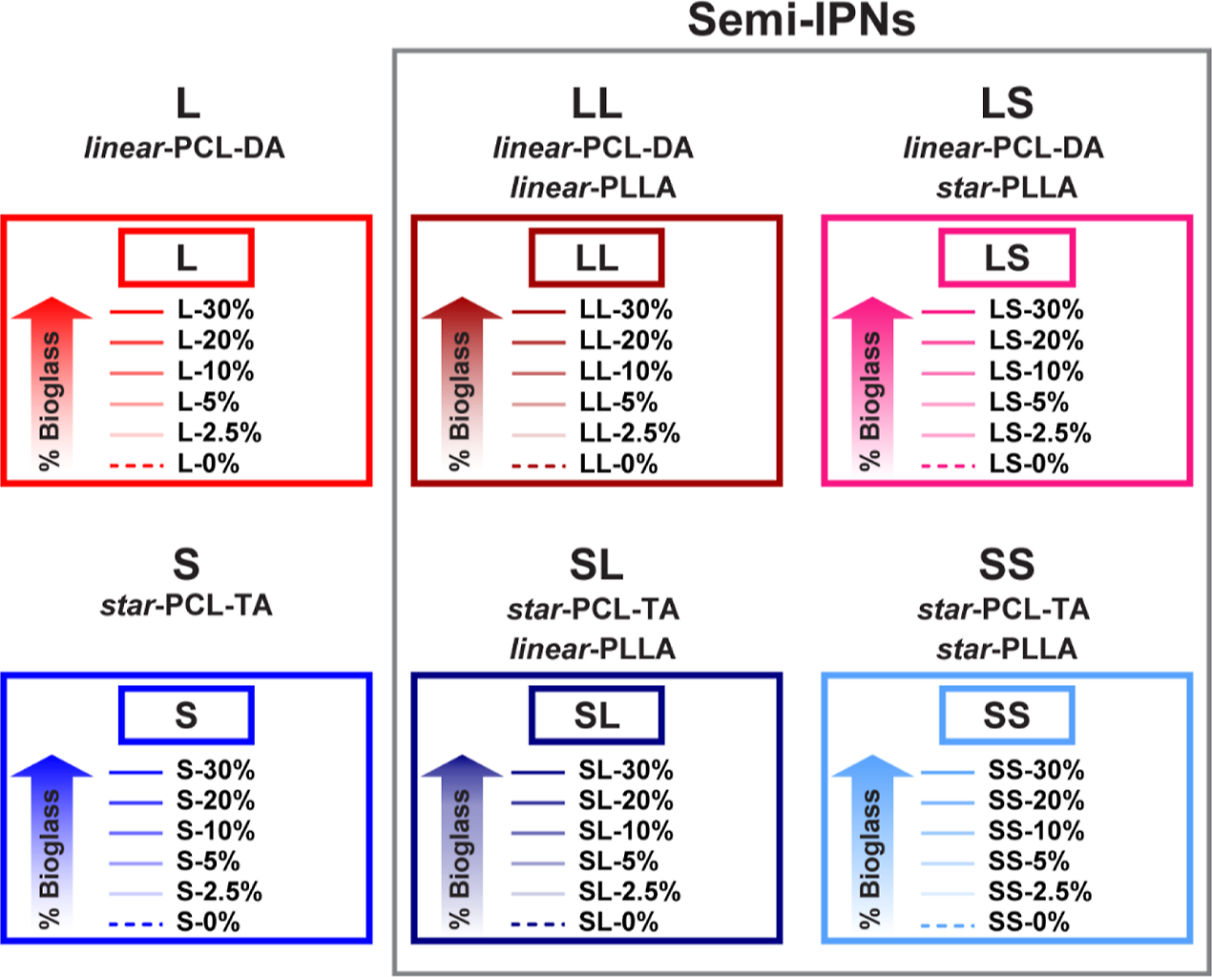
Composite scaffold compositions and designations (e.g., “L-5%). PCL-based macromers were used alone, and in combination with PLLA (to form semi-IPNs). The architecture (*linear* or *star*) was varied for both the PCLs and PLLAs. Percentages signify wt % of 45S5 BG incorporated with respect to total polymer.

Herein, toward creating potently bioactive, self-fitting SMP scaffolds, BG was introduced to form composite scaffolds. Fabrication was accomplished by direct incorporation of the BG to the salt template. This yielded “BG templated” composite scaffolds with BG concentrated at the pore wall surfaces and minimized within the pore wall struts. Both the BG level and macromer composition were systematically tuned to create a library of composite scaffolds (Figure 1). *Linear*-PCL-DA (L) and *star*-PCL-TA (S) were utilized alone and, for semi-IPNs, in combination with *linear*- and *star*-PLLA. The macromer solution makeup for semi-IPNs consisted of PCL/PLLA (3:1 wt %) with *linear*-PCL-DA/*linear*-PLLA (LL), *linear*-PCL-DA/*star*-PLLA (LS), *star*-PCL-TA/*linear*-PLLA (SL) and *star*-PCL-TA/*star*-PLLA (SS). BG levels were systematically varied at 0, 2.5, 5, 10, 20, and 30 wt % with respect to the macromers. Thermal gravimetric analysis (TGA), Alizarin Red S staining, and scanning electron microscopy (SEM) were used to assess the BG incorporation into the scaffolds. The resulting composite scaffolds were subjected to comprehensive analyses of material properties (e.g., sol content, pore size, pore interconnectivity, and compressive mechanical properties). Selected compositions were further evaluated based on thermal properties (e.g., *T*_m_, % crystallinity), degradation profiles, shape memory, and bioactivity.

## 2 Materials and Methods

### 2.1 Materials

*Linear*-PCL-diol (PCL-diol, *M*_n_ = 10k g mol^–1^ per manufacturer), 4-dimethylaminopyridine (DMAP), triethylamine (Et_3_N), acryloyl chloride, potassium carbonate (K_2_CO_3_), anhydrous magnesium sulfate (MgSO_4_), sodium chloride (NaCl), (3S)-*cis*-3,6-dimethyl-1,4-dioxane-2,5-dione (l-lactide), ε-caprolactone, ethylene glycol, pentaerythritol, tin(II) 2-ethylhexanoate (Sn(Oct)_2_), *N*-vinyl-2-pyrrolidone (NVP), 2,2-dimethoxy-2-phenylacetophenone (DMP), deuterated chloroform (CDCl_3_), sodium hydroxide (NaOH), phosphate-buffered saline (PBS, pH 7.4), sodium bicarbonate (NaHCO_3_), potassium chloride (KCl), potassium phosphate dibasic trihydrate (K_2_HPO_4_·3H_2_O), magnesium chloride hexahydrate (MgCl_2_·6H_2_O), hydrochloric acid (HCl), calcium chloride (CaCl_2_), sodium sulfate (Na_2_SO_4_), tris(hydroxymethyl)-aminomethane (tris), Alizarin Red S, acetic acid, and solvents were purchased from Sigma-Aldrich. All solvents and ethylene glycol were dried over 4 Å molecular sieves prior to use. 45S5 BG (10 μm) was purchased from Mo-Sci Corp (Rolla, MO, USA).

### 2.2 Syntheses

All reactions were run in glassware with Teflon-covered stir bars, which were dried at 120 °C prior to use. Reactions took place in a positive nitrogen (N_2_) environment. After synthesis and purification, polymer structures [including *M*_n_ and % acrylation (>90%)] were confirmed with ^1^H NMR spectroscopy (400 MHz spectrometer operating in Fourier Transform mode with CDCl_3_ as the standard).

*Star*-PCL-tetrol (*M*_n_ = 10 kg mol^–1^) was synthesized via ring opening polymerization according to an established procedure.^22^ Briefly, ε-caprolactone (20.0 g), pentaerythritol (0.278 g, 88:1, [M]:[I]), and Sn(Oct)_2_ were combined in a round-bottom (rb) flask and allowed to react overnight (ON) at 120 °C. The crude product was dissolved in CH_2_Cl_2_, precipitated in cold CH_3_OH, and vacuum-dried [room temperature (RT), ON, 30 in. Hg] to yield a purified *star*-PCL-tetrol. The ^1^H NMR spectrum agreed with that previously reported.^22^

*Linear*-PCL-diol (*M*_n_ = 10 kg mol^–1^) and *star*-PCL-tetrol (*M*_n_ = 10 kg mol^–1^) were acrylated to produce *linear*-PCL-diacrylate (DA) and *star*-PCL-tetraacrylate (TA), respectively, per prior reports.^20,22^*Linear*-PCL-diol (20.0 g, 2.0 mmol) and DMAP (6.6 mg) were dissolved in dichloromethane (DCM, 0.17 g mL^–1^). The rb flask was purged for 3 min with N_2_, and Et_3_N (4.0 mmol) and acryloyl chloride (8.0 mmol) were subsequently added dropwise. The reaction was allowed to proceed under positive N_2_ pressure for 30 min, followed by reflux for 16 h at 55 °C. Solvent was removed via rotary evaporation and the crude product was dissolved in ethyl acetate (135 mL) and gravity filtered. Solvent was once again removed, PCL redissolved in DCM (140 mL), washed with 13.5 mL K_2_CO_3_, and the layers allowed to separate in a separatory funnel. The organic layer was collected, dried with MgSO_4_, filtered, and the solvent was removed via rotary evaporation. Finally, the product was dried under vacuum (RT, ON, 30 in. Hg) to yield purified *linear*-PCL-DA. A similar procedure was performed for the *star*-PCL-tetrol acrylation, but with doubled molar ratios to account for the 4-armed polymer (DMAP = 13.2 mg, Et_3_N = 8.0 mmol, acryloyl chloride = 16.0 mmol). ^1^H NMR spectra agreed with that previously reported.^22^

*Linear*- and *star*-PLLA (*M*_n_ ∼ 15k g mol^–1^) were synthesized per a prior report.^28^ Briefly, for *linear*-PLLA, l-lactide (6.0 g), ethylene glycol (25 mg), and Sn(Oct)_2_ were combined and reacted ON at 120 °C. For *star*-PLLA, pentaerythritol (54.5 mg) was used in place of ethylene glycol. The reactions were quenched with removal from heat and exposure to O_2_. The crude *linear*- or *star*- PLLA was dissolved in chloroform and precipitated into cold CH_3_OH. The purified products were filtered and dried under vacuum (RT, ON, 30 in. Hg). ^1^H NMR spectra agreed with that previously reported.^22^

### 2.3 Fabrication of Scaffolds

Scaffolds were fabricated via SCPL, and the manner of BG introduction was initially evaluated with scaffolds prepared exclusively from *linear*-PCL with 0 to 30 wt % BG (Figure S1). The “reduced centrifuge” method mimicked the prior SCPL protocol (i.e., “unmodified”)^20^ to prepare SMP scaffolds. However, BG was introduced to the DCM-based macromer solution, but subsequently centrifuged with reduced time (2 min vs 10 min) through the salt template. The “glass-in-salt mold” method involved incorporation of the BG into the salt template, and macromer centrifugation was maintained per the “unmodified” protocol.

**Figure 2:**
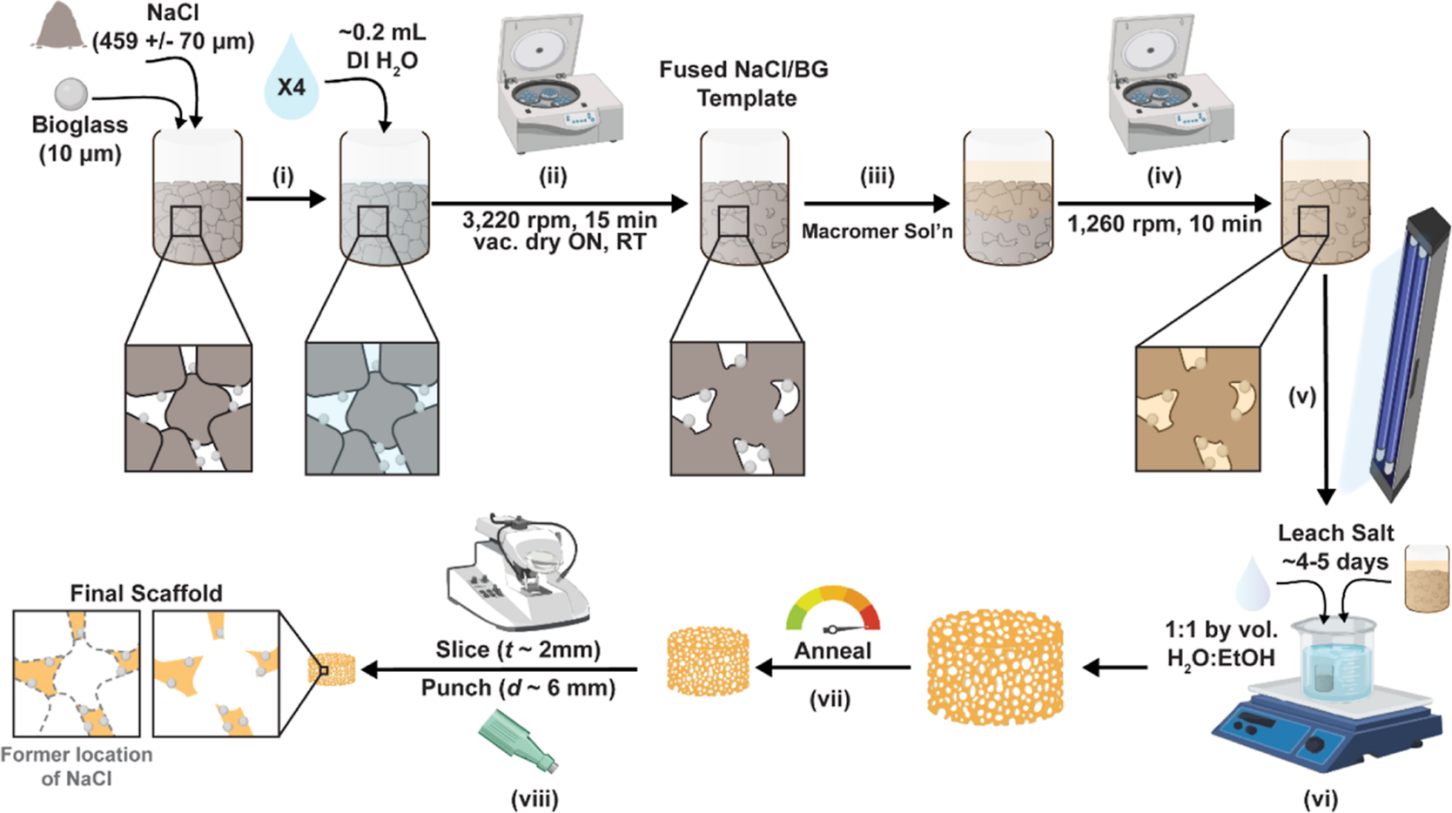
SCPL “glass-in-salt mold” fabrication of composite scaffolds: (i) BG and salt (NaCl) were combined in a scintillation vial and mechanically mixed; (ii) DI H_2_O was added and the vial centrifuged to create a fused BG/salt template; (iii) the designated macromer solution was added to the fused BG/NaCl template; (iv) the vial was centrifuged to diffuse the solution throughout the template; (v) the vial was UV-cured for ∼7 min; (vi) the salt was leached by soaking in a H_2_O/EtOH solution; (vii) the scaffold was annealed; (viii) and the scaffold was sliced and punched to yield final specimens. This fabrication method yielded composite scaffolds with BG concentrated at pore walls.

(i) “Reduced centrifuge”: Sieved NaCl (10 g, 459 ± 70 μm) was added to a 20 mL scintillation vial (I.D. = 25 mm). Deionized (DI) H_2_O (7.5 wt %) was added in four portions with mixing after each addition with a spatula. The wet salt mold was packed down with a plastic rod and centrifuged (3220 rpm, 15 min). Next, the vials were uncapped and vacuum-dried (RT, ON, 30 in. Hg). BG was incorporated into the macromer solutions at designated wt % (based on total macromer wt). Macromer solutions were prepared by dissolving the polymer(s) in DCM (0.15 g mL^–1^), and suspending the BG in DCM via continuous stirring at 500 rpm. Photoinitiator solution (10 wt % DMP in NVP) was added to the macromer solution at 15 vol % and mixed on a shaker plate. Approximately 5 mL of the macromer solution was added to each salt template and the vials were capped and centrifuged (1260 rpm, 2 min) to diffuse the macromer solution through the salt template. Next, the vials were uncapped and placed on a UV plate for ∼7 min to cross-link the acrylated PCL (UV-Transilluminator, 6 mW cm^–2^, 365 nm). After UV exposure, the vials were air-dried in a fume hood ON followed by 5 h of drying under vacuum (RT, 30 in. Hg). The NaCl was then removed by placing the vials in a water and ethanol (EtOH) solution (1:1 by vol) for ∼5 days with daily solution changes. The resulting scaffolds were then air-dried (∼48 h) and annealed under vacuum (30 in. Hg) at 85 °C (1 h for non-semi-IPNs) or 180 °C (5 min for semi-IPNs). After 48 h, each resulting cylindrical specimen (*d* ∼ 12 mm) was sliced into discs (*t* ∼ 2 mm) (Vibratome, Leica VT 1000 S), disregarding the top and bottom of the original specimen. Finally, the discs were biopsy punched (Integra Miltex, 6 mm) to yield scaffold specimens (*d* ∼ 6 mm × *t* ∼ 2 mm).
(ii) “Glass-in-salt mold”: Sieved salt (10 g) was added to the scintillation vials, and the designated wt % BG (based on total macromer wt) was added into the vial with the salt and mixed with a spatula. After each addition of H_2_O (7.5 wt %) to the salt and BG, vigorous mechanical stirring was performed to evenly disperse BG throughout the salt mold template. The NaCl/BG template was centrifuged (3220 rpm, 15 min) to fuse the template. After drying (RT, ON, 30 in. Hg) the fused template, macromer solutions were prepared by dissolving the polymer(s) in DCM (0.15 g mL^–1^). The photoinitiator solution (DMP dissolved in NVP) was added to the macromer solution as previously described. Finally, the macromer solution was added to the NaCl/BG template, centrifuged (1260 rpm, 10 min), cured, dried, annealed, and sectioned as previously noted to produce scaffold specimens (*d* ∼ 6 mm × *t* ∼ 2 mm) (Figure 2).

### 2.4 Fabrication of Solid Films

Analogous films were prepared for scaffold porosity calculations. The macromer solutions with designated wt % BG were stirred at 500 rpm ON in a rb flask, and the aforementioned photoinitiator solution (15 vol %) added with vortexing. The mixture was transferred to a silicone mold (*d* ∼ 50 mm × *t* ∼ 2 mm, McMaster-Carr) secured between 2 glass slides. The mold was then placed over UV light (UV-Transilluminator, 6 mW cm^–2^, 365 nm) for 6 min, flipping halfway through. The resulting films were allowed to air-dry ON, soaked in EtOH over a shaker plate (150 rpm, 3 h), air-dried ON, and annealed at 85 °C (1 h for non-semi-IPNs) or 180 °C (5 min for semi-IPNs). Finally, a 6 mm biopsy punch was used to cut out specimens.

### 2.5 Scaffold Characterization

#### 2.5.1 Thermal gravimetric analysis

TGA (TA Instruments Q50) was performed using scaffold specimens (*N* = 3) [with diameters reduced to 4 mm via a biopsy punch] from RT to 600 °C in platinum pans (N_2_ environment, heating rate of 10 °C min^–1^).

#### 2.5.2 Sol Content

Scaffold specimens (*N* = 3) were submerged in 10 mL of DCM in a scintillation vial and placed atop a shaker plate (150 rpm, 48 h). Scaffolds were then removed, rinsed with DCM, allowed to air-dry ON in a fume hood, and dried under vacuum (RT, ON, 30 in. Hg). Scaffold initial and final masses were used to determine sol content.

#### 2.5.3 Pore Size, % Porosity, and Pore Interconnectivity

Pore size was evaluated via SEM (Tescan Vega 3, accelerating voltage of 10 kV). Cross sections of scaffold specimens were coated with Au–Pt (∼10 nm). The average pore size was determined from measurements (*N* = 10) of pores measured along the diagonal midline with ImageJ software. The % porosity of scaffolds (*N* = 3) was determined by eq 1.^29^ Pore interconnectivity (*N* = 3) was evaluated on scaffolds using a water-wicking test.^30^ Briefly, specimens were submerged in DI water and placed on a shaker plate for 24 h (150 rpm). Scaffolds were then removed from the water and weighed (mass_total_). The scaffold was weighed once again to obtain mass_interconnected_ after a Kimwipe was used to wick away interconnected water. Pore interconnectivity was calculated with eq 2.
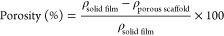
1
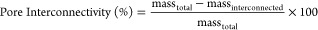
2

#### 2.5.4 Alizarin Red S Staining

Alizarin Red S staining was used to stain for calcium of BG to confirm BG incorporation. Alizarin Red S was dissolved in DI H_2_O (2% w/v) and the pH was adjusted to 4.2. In well plates containing the scaffolds, the solution was added, the scaffolds were soaked for 5 min, and then rinsed with DI water to remove non-specific staining. For quantification, the stained scaffolds were soaked in a solution of 10% acetic acid and 20% CH_3_OH in water on a shaker plate (150 rpm, 15 min), and the solution was read on a spectrophotometer (Cytation 5 Biotek) at 450 nm after removal of the scaffolds from the well plate.

#### 2.5.5 Compression Testing

Compressive mechanical properties were evaluated at RT with an Instron 5944 (2 kN load cell). Scaffold specimens (*N* = 5) were subjected to an initial preload force of 0.1 N, and the strain zeroed. A constant compressive strain rate of 1.5 mm min^–1^ was applied until 85% strain. From the resulting stress–strain curves, the compressive modulus (*E*) was determined from the slope of the initial linear region (0–10% strain). Compressive strength (*CS*) was determined from the stress at 85% strain. Toughness was calculated from the integration of the stress–strain curve from 0 to 85% strain. *E*, *CS*, and toughness were all calculated using a custom MATLAB code.

#### 2.5.6 *T*_m_ and % Crystallinity

PCL and PLLA *T*_m_ and % crystallinity in scaffolds were determined by differential scanning calorimetry (DSC, TA Instruments Q100). Samples harvested from scaffolds (∼10 mg, *N* = 3) were sealed in hermetic pans and heated at 5 °C min^–1^. All values were obtained from the second heating cycle to erase thermal history. The *T*_m_ was determined from the maximum point on the endothermic melt peak for PCL and PLLA, and percent crystallinity was determined with eq 3.
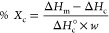
3where Δ*H*_m_ is the enthalpy of fusion from the endothermic melt peak, Δ*H*_c_ is the enthalpy from the exothermic cold crystallization peak, Δ*H*_c_^°^ is the enthalpy of fusion of theoretical 100% crystalline PCL (139.5 J g^–1^)^31^ or PLLA (93.0 J g^–1^),^32^ and *w* is the mass fraction of the respective polymer.

#### 2.5.7 Shape Memory Properties

##### 2.5.7.1 Qualitative Shape Memory: “Self-Fitting” into an Irregular Model Defect

Scaffold specimens (*N* = 3) were evaluated for their “self-fitting” properties into an irregular defect. An “irregular defect” was created in an ultra-high-molecular-weight polyethylene (UHMWPE) sheet with a drill press (Grizzly G7948). This irregular defect possessed a max height and width of ∼5.65 × 5.35 mm. The scaffolds were submerged in water for 1 min at a temperate 5 °C higher than the *T*_m_ (i.e., 60 °C for scaffolds based on *linear*-PCL [L, LL, LS], or 50 °C for scaffolds based on *star*-PCL [S, SL, SS]). The scaffolds were then press-fit into the irregular defect, and allowed to cool to RT for 3 min. After cooling, the scaffold was taken out of the model defect in its shape-fixed state. Finally, the scaffold was submerged in the warm water once again and allowed to recover to its original shape. Photographs were taken at each stage of this process.

##### 2.5.7.2 Quantitative Shape Memory: Shape Fixity (*R*_f_) and Recovery (*R*_r_) into a Circular Defect

Scaffold specimens (*N* = 3) were evaluated based on a previously developed procedure.^22^ A model circular defect (∼5 mm) was created using an UHMWPE sheet with a drill press. The initial diameter of the scaffolds were measured, followed by submersion for 1 min in a water bath as noted above. The scaffold was removed from the water bath, and immediately press-fit into the model defect and allowed to cool to RT for 3 min. After cooling, the scaffold diameter was measured in its “fixed shape.” Scaffolds were resubmerged in the water bath (∼1 min), removed, and allowed to cool to RT. The diameter of the scaffold was measured to assess the “shape recovery.” The procedure was repeated once more to determine *R*_f_ and *R*_r_ for 2 cycles. Reported values used the second cycle to eliminate thermal history that may be present for cycle 1. The *R*_f_ and *R*_r_ for cycle 1 (*N* = 1) and cycle 2 (*N* = 2) were calculated using the following equations
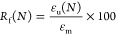
4
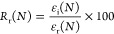
5where ε_u_(*N*) is the scaffold diameter after being press-fit into the mold, ε_m_(*N*) is the diameter of the mold, ε_*i*_(*N*) is the diameter after shape-recovering from the mold, and ε_*r*_(*N*) is the initial diameter of the scaffold. All diameters were measured via a caliper with a resolution of 0.01 mm.

#### 2.5.8 Degradation

##### 2.5.8.1 Accelerated Degradation

Scaffold specimens (*N* = 3 per time-point) were subjected to base-catalyzed conditions (0.2 M NaOH) per ASTM F1635.^33^ Specimens were each submerged in 10 mL of the NaOH solution, sealed in a 20 mL glass scintillation vial, and placed in an incubator (VWR Benchtop Shaking Incubator model 1570, 37 °C, 60 rpm). At daily time points for 7 days, samples were removed, rinsed with DI water, and dried under vacuum (RT, 48 h, 30 in. Hg). The mass of the dried scaffold was compared to the initial mass to calculate % mass loss. All scaffolds were only used for a single time-point.

##### 2.5.8.2 Non-accelerated Degradation

Scaffold specimens (*N* = 3 per time-point) were submerged in 10 mL PBS (pH = 7.4), sealed in 20 mL glass scintillation vials, and placed in an incubator (VWR Benchtop Shaking Incubator model 1570, 37 °C, 60 rpm). At 3, 5, 6, 7, 8, 9, 10, and 12 months, the scaffolds were removed from the PBS solution, rinsed with DI water, and dried under vacuum (RT, 48 h, 30 in. Hg). The mass of the dried scaffold was compared to the initial mass to calculate % mass loss. All scaffolds were only used for a single time-point.

#### 2.5.9 Bioactivity

SBF (1×) was prepared as described by Kokubu.^34^ Scaffold specimens (*N* = 3 per-time-point) were each placed in a sealed centrifuge tube containing ∼10 mL of SBF and submerged in a water bath at 37 °C. At 1 day and 2 week time points, scaffolds were removed from the solution, rinsed with DI water, and dried under vacuum (RT, ON, 30 in. Hg). Specimens were coated with Au–Pt (∼10 nm) and analyzed using SEM (Tescan Vega 3, accelerating voltage of 10 kV) to visualize HAp mineralization. Energy-dispersive X-ray spectroscopy (EDS, Oxford Instruments) was utilized to determine elemental molar composition of suspected HAp mineralization.

### 2.6 Statistical Analyses

Values were compared in GraphPad Prism via 2-way ANOVA tests, where a *p*-value of <0.05 was considered statistically significant. Data was reported as a mean ± standard deviation.

## 3 Results and Discussion

### 3.1 Scaffold Fabrication and Characterization

To evaluate an effective method to incorporate BG at controlled wt % levels, composite scaffolds based on *linear*-PCL (L-0%, L-2.5%, L-5%, L-10%, L-20%, and L-30%) were first prepared via three distinct protocols (Figure S1). Previously, SMP scaffolds were effectively formed via SCPL employing a salt template that was fused by the addition of water, and a DCM-based macromer solution subsequently cast over the template with centrifugation (1260 rpm, 10 min) employed to assist diffusion (i.e., “unmodified” method).^20^ However, introducing BG directly into the macromer solution resulted in its separation during centrifugation. This was improved by reducing the centrifuge time (2 min) (i.e., “reduced centrifuge” method). Alternatively, the BG was incorporated into the salt template by combining the salt, BG, and water to effectively form a BG/fused salt template (i.e., “glass-in-salt mold” method). Subsequently, the DCM-based macromer solution was cast over this template in the manner previously used (1260 rpm, 10 min). Composite scaffolds fabricated with these two methods were subjected to TGA to reveal the resulting wt % BG content based on wt % remaining at 600 °C (Figure S2 and Table S1). Overall, the “glass-in-salt mold” method resulted in BG wt % levels with improved reproducibility and alignment with targeted values. Furthermore, as described below, the “glass-in-salt mold” method was expected to yield composite scaffolds with the BG concentrated on the pore wall surface rather than within the pore struts. This was subsequently predicted to contribute to enhanced scaffold bioactivity through the exposure of BG to the physiological environment. Moreover, a lack of BG within the struts was anticipated to avoid brittleness.

The entire series of SMP composite scaffolds (Figure 1 and Table S2) was subsequently formed by SCPL with the “glass-in-salt mold” method (Figure 2). Sol content results showed that the presence of BG did not inhibit PCL cross-linking within composite scaffolds (Figure S3 and Table S3). For non-semi-IPN composites (i.e., no PLLA), sol content values remained similarly low (<10%) as compared to analogous scaffolds with no BG. Semi-IPN scaffolds without BG exhibited sol contents < ∼ 28%, attributed to the presence of un-cross-linked PLLA (75:25 wt % PCL/PLLA). With the introduction of BG, sol content values decreased with increasing wt % BG, perhaps due to the diminished ability to extract the PLLA.

**Figure 3:**
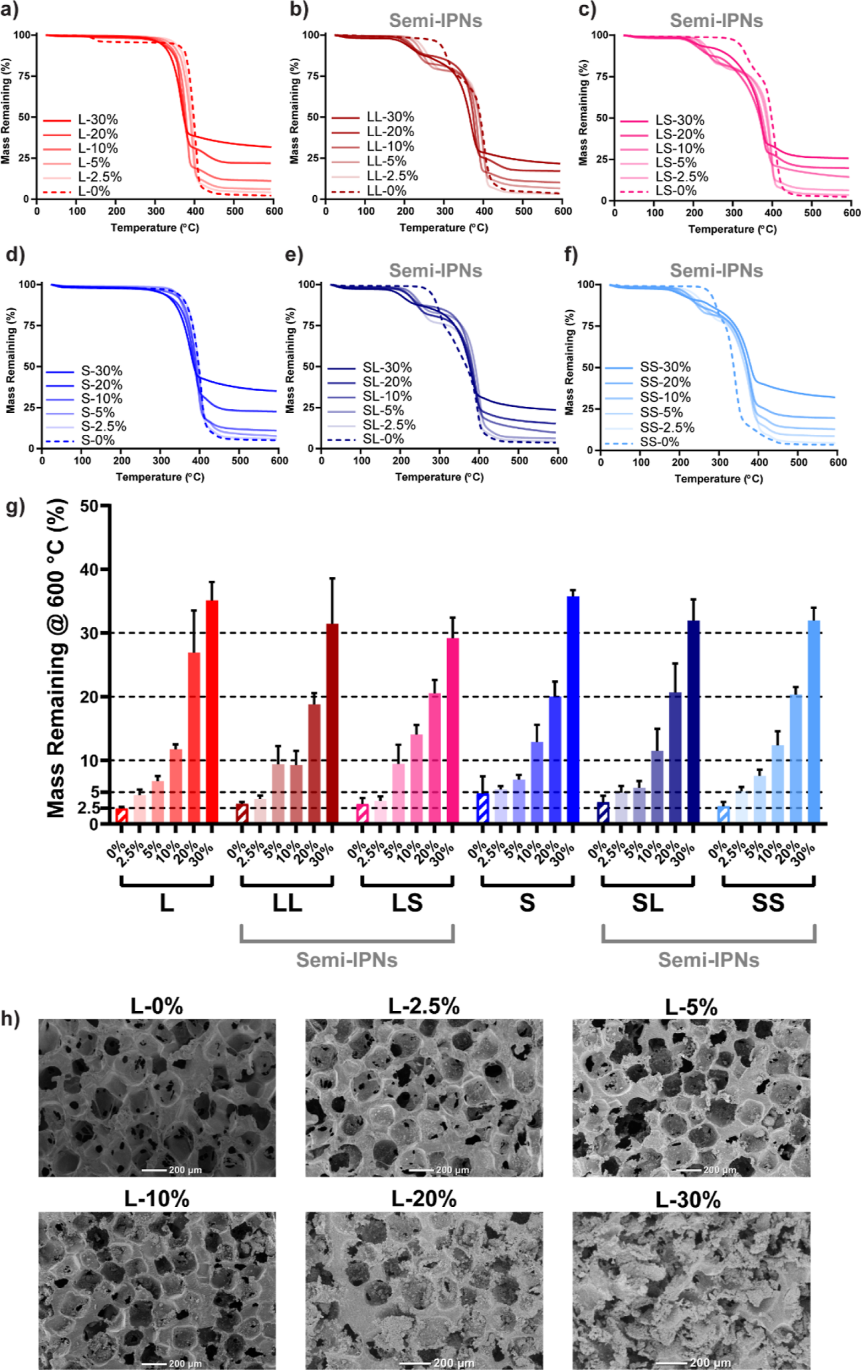
TGA of (a) L, (b) LL, (c) LS, (d) S, (e) SL, and (f) SS scaffold series. (g) wt % plateau values at 600 °C, corresponding to wt % BG; “---” line indicates wt % BG used for scaffold fabrication. (h) SEM images of L scaffold series.

The incorporation of BG wt % near targeted levels was likewise confirmed with TGA (Figure 3a–g and Table S4). For semi-IPN compositions, an initial mass loss of ∼25 wt % (∼250–300 °C) corresponded to PLLA degradation within the cross-linked PCL network as noted previously.^35^

The presence and distribution of BG, as well as its impact on composite scaffold morphology was assessed. SEM analysis showed the successful leaching of NaCl and the presence of BG at increasing levels based on the wt % introduced during fabrication (Figure 3h). SEM images also revealed that the BG was concentrated on the pore wall surface, rather than within the pore struts (Figure S4). This BG distribution was associated with the BG/fused salt template used in the “glass-in-salt mold” method. Composite scaffolds also exhibited good pore interconnectivity (∼40–60%) (Figure S5 and Table S5). Scaffolds without BG exhibited pore sizes ∼250 μm, within the range (200–300 μm) known to promote bone regeneration.^36,37^ Composite scaffolds with ≤10 wt % BG exhibited similar pore sizes (Figure S6 and Table S5). However, as BG increased to 20 and 30 wt %, a reduction in pore wall integrity was observed due to “overloading” with BG. This resulted in somewhat higher pore sizes. As a result, % porosity (∼60–70%) was somewhat varied for composite scaffolds with >10 wt % BG (Figure S7 and Table S5). The identity of BG (owing to the constituent calcium) was confirmed with Alizarin Red S staining with select compositions comprised of 0, 5, and 10 wt % BG (Figure S8 and Table S6). While scaffolds without BG generally exhibited absorbances of ∼0.2, composite scaffolds exhibited absorbances that increased (∼0.3–0.75) as the wt % of BG increased.

### 3.2 Mechanical Properties

**Figure 4:**
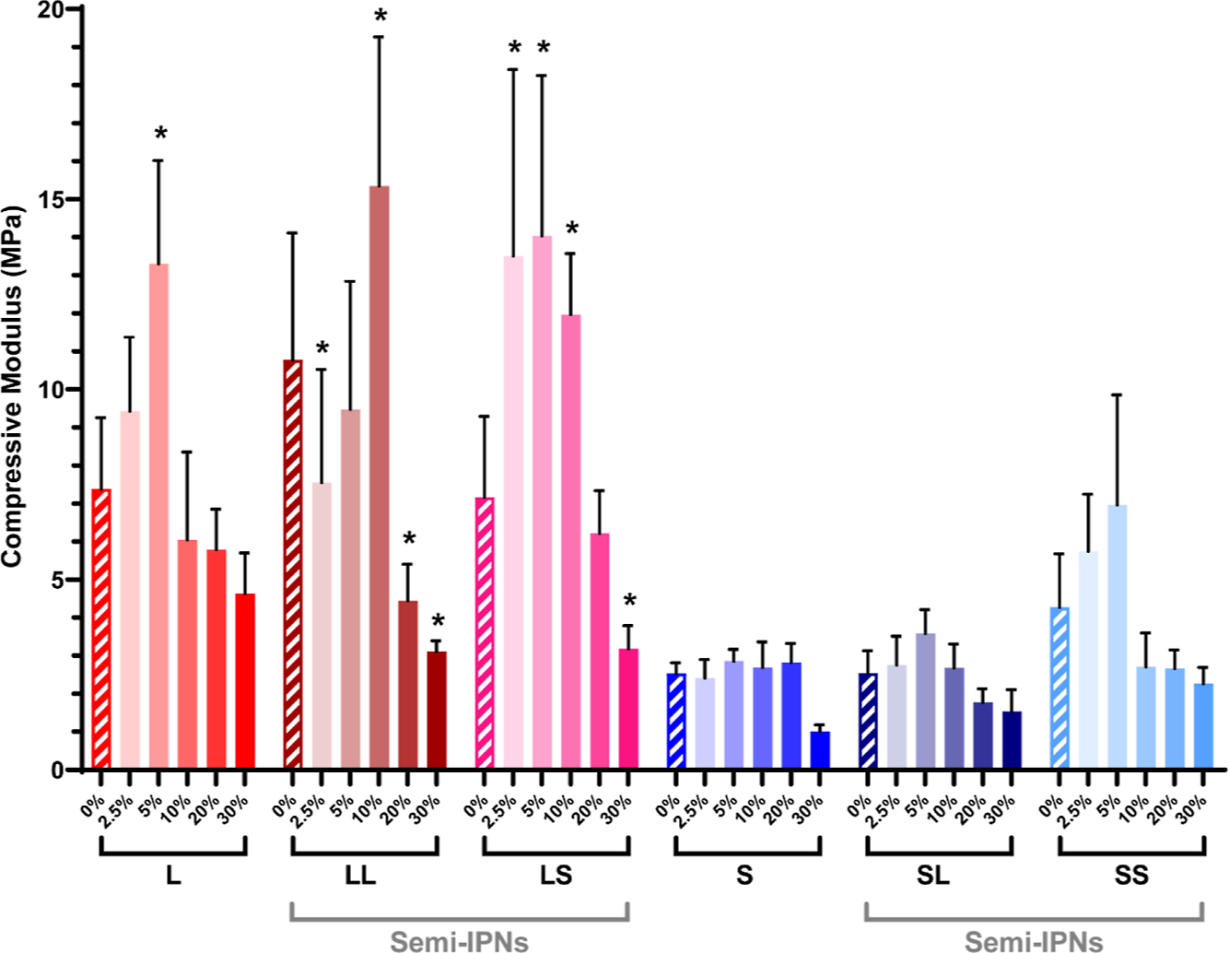
Scaffold compressive modulus values; *p < 0.05 vs 0% BG of analogous macromer composition.

Mechanically robust scaffolds are essential to their utility to heal bone defects.^38^ The impact of BG on scaffold mechanical properties was assessed by static compression testing. From the resulting stress–stain curves (Figure S9), *E* (Figure 4), *CS* (Figure S10a), and toughness (Figure S10b) were measured (Table S7). The composition of the matrix as well as BG levels had significant impact on these properties. In terms of modulus, scaffolds based on *linear*-PCL were more rigid than those based on *star*-PCL. This is attributed to the higher % crystallinity of *linear*-PCL (as described below). For BG-free semi-IPN scaffolds based on *linear*-PCL, *E* increased more with the inclusion of *linear*-PLLA versus with *star*-PLLA (LL-0% > LS-0% ≈ L-0%). In the case of BG-free semi-IPN scaffolds based on *star*-PCL, the incorporation of *star*-PLLA led to a higher *E* (SS-0% > SL-0% ≈ S-0%). Few composite scaffolds exhibited significantly higher *E* versus corresponding non-BG scaffolds; these notably contained low BG levels (≤10 wt %): L-5%, LL-10%, LS-2.5%, and LS-5%. In fact, with higher (>10 wt %) BG levels, composite scaffolds generally exhibited a reduction in *E*. This is attributed to the diminished pore wall integrity with higher BG levels (Figure 3h). Likewise, composite scaffold *CS* and toughness were generally maximized for those with 5 or 10 wt % BG. Importantly, all scaffolds exhibited non-brittle behavior and did not fracture during testing (i.e., withstood 85% strain). The concentration of BG at the pore wall surfaces is hypothesized to favorably avoid brittleness.

### 3.3 Thermal Properties

**Figure 5:**
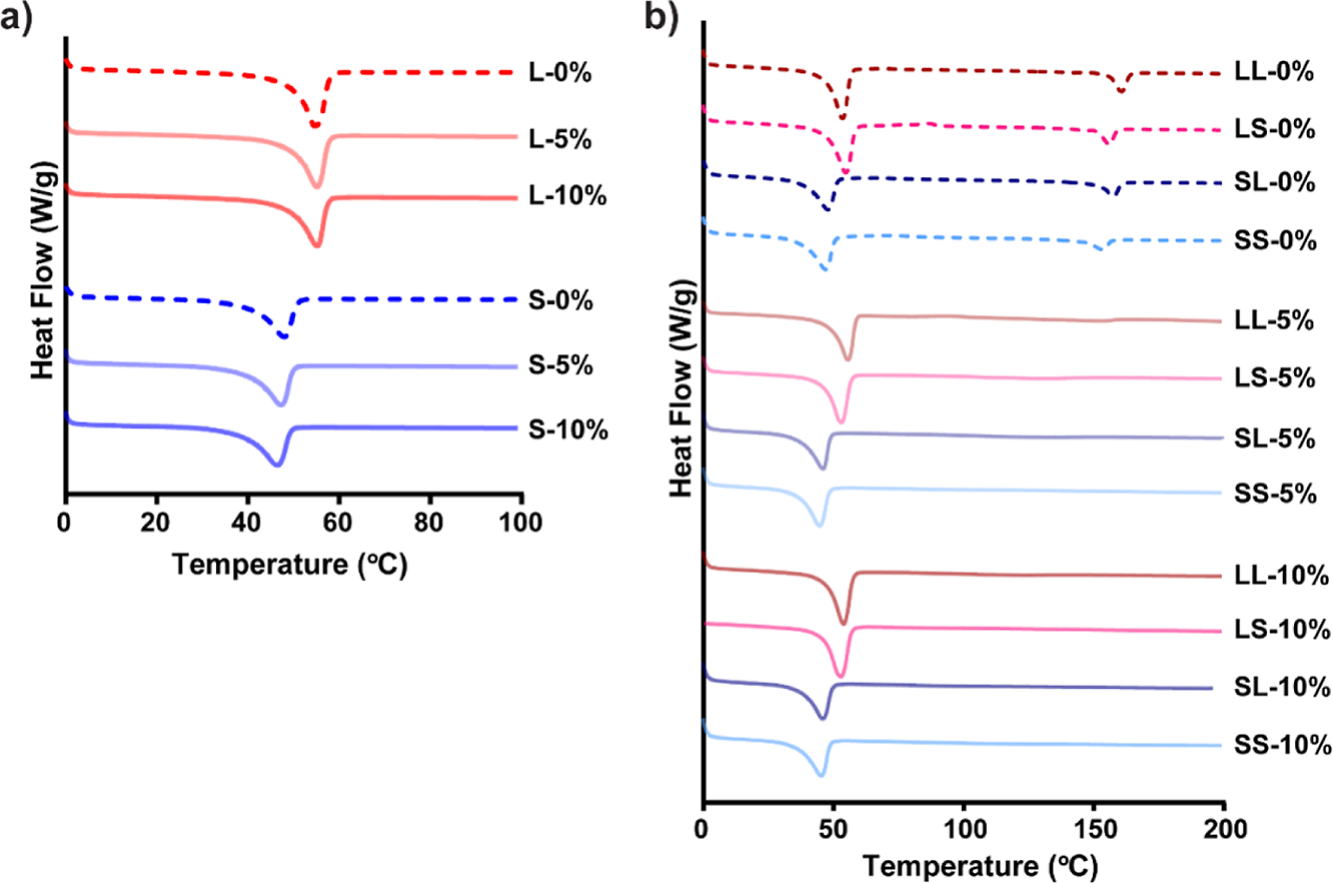
Representative thermograms for (a) non-semi-IPN scaffolds and (b) semi-IPN scaffolds.

The *T*_m_ and % crystallinity of scaffolds were evaluated because of their potential impact on not only shape memory properties (wherein *T*_m,PCL_ serves as the shape transition temperature), but also mechanical and degradation properties. Scaffolds with lower BG levels (i.e., 0, 5, 10 wt %) were evaluated given the previously noted limitations of those containing 20 and 30 wt % BG. *T*_m,PCL_ was maintained as was generally % crystallinity for all scaffolds based on *linear*-PCL (∼55 °C; ∼32–42%) or *star*-PCL (∼45 °C; ∼28–35%), irrespective of BG levels (Figures 5a, S11a,c and Table S8). The contributions of higher PCL % crystallinity to greater *E* values are notable (Figure 4). For non-BG scaffolds, the higher % crystallinity of *linear*-PCL (L-0%; ∼42%) versus *star*-PCL (S-0%; ∼31%) contributed to the greater *E*, despite the relatively lower cross-link density. For semi-IPN scaffolds, the *T*_m,PLLA_ and % crystallinity of PLLA were also analyzed (Figures 5b, S11b,d and Table S8). For non-BG scaffolds, *T*_m,PLLA_ and % crystallinity were maintained between ∼152–162 °C and ∼30–48%, respectively. PLLA % crystallinity was highest for LL-0% and lowest for SS-0%, consistent with our prior report.^22^ However, with the addition of BG to semi-IPN scaffolds, PLLA crystallinity was completely lost. A reduction in polyester crystallinity when combined with bioceramics has been previously observed.^39^ We attribute the loss of PLLA crystallinity, yet retention of PCL crystallinity, to the relatively enhanced interaction of less hydrophobic PLLA chains (relative to PCL) with the BG that inhibits crystallization. Despite the loss of PLLA crystallinity, composite semi-IPN scaffolds (5 and 10 wt % BG) exhibited similar mechanical properties versus analogous non-BG scaffolds.

### 3.4 Shape Memory Properties

Scaffold *R*_f_ and *R*_r_ were evaluated for scaffolds with 0, 5, and 10 wt % BG. For qualitative tests, after exposure to warm water (60 °C for *linear*-PCL based scaffolds, 50 °C for *star*-PCL based scaffolds), all specimens successfully fit into the irregular model defect (Figure S12). After cooling to RT, the scaffolds were locked into their new shape, and maintained their shapes even after removal from the mold (i.e., *R*_f_). Once exposed again to the warm water, all scaffolds displayed the ability to recover (i.e., *R*_r_) to their original shapes. Quantitative shape memory tests revealed that all *R*_f_ and *R*_r_ values (2nd cycle) were near 100% for all scaffold compositions (Table S9). The excellent shape memory behavior of composite scaffolds is attributed to sufficient retention of PCL crystallinity.

### 3.5 Degradation Profiles

**Figure 6:**
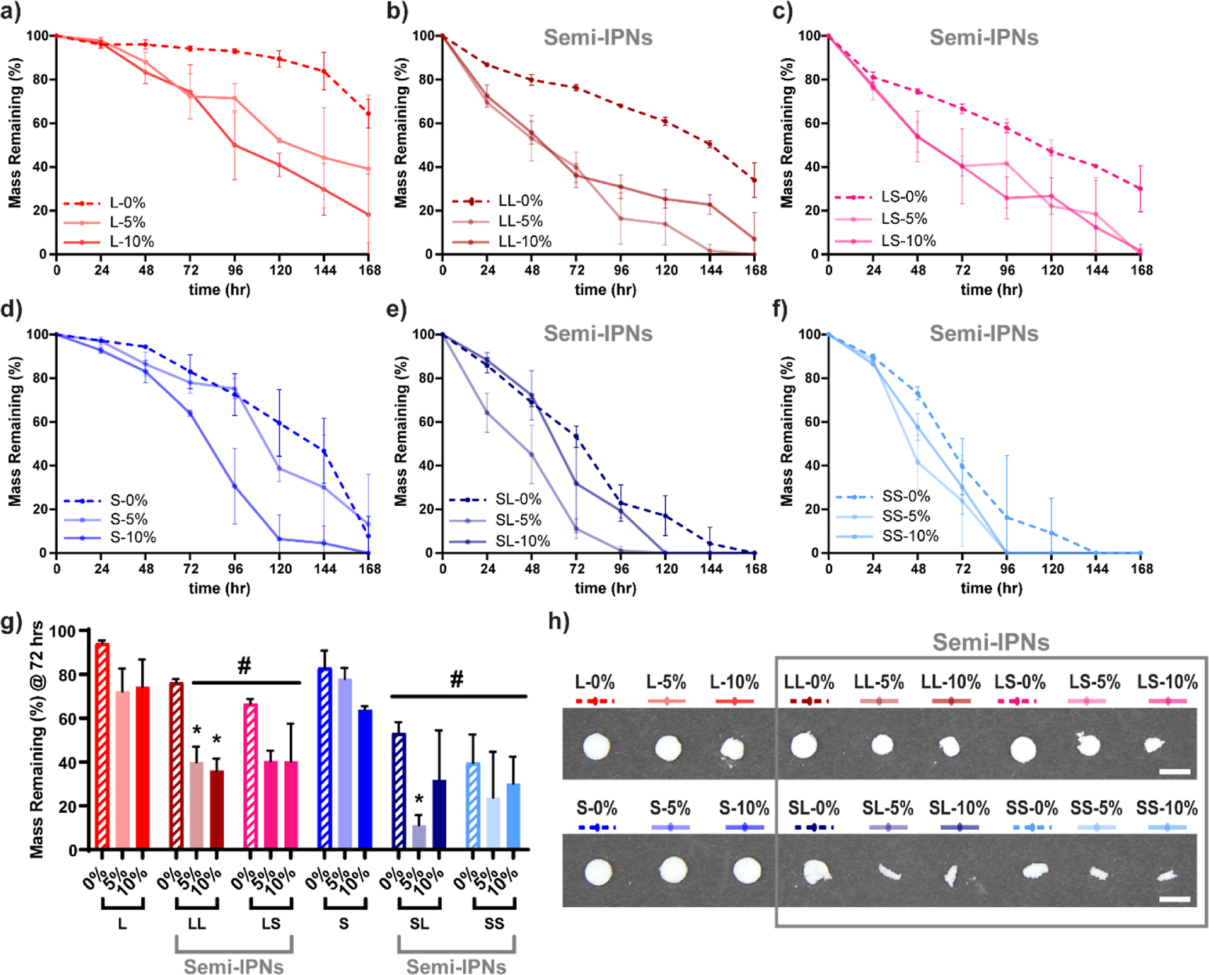
Mass loss over time for accelerated degradation studies (0.2 M NaOH, 37 °C, 60 rpm) for (a) L, (b) LL, (c) LS, (d) S, (e) SL, (f) SS scaffolds. (g) Mass remaining of scaffolds at 72 h; *p < 0.05 vs 0% BG of analogous macromer composition; #*p* < 0.05 compared against same BG % of analogous architecture PCL non-semi-IPN. (h) Photoseries of scaffolds at 72 h of accelerated degradation study (scale bars = 6 mm).

Scaffold degradation rates that better align with neotissue formation are expected to improve bone defect healing.^16^ We previously reported that PCL–PLLA semi-IPN scaffolds degraded significantly faster than PCL scaffolds, attributed to phase separation that increased water permeability.^22^ Incorporation of bioceramics has been shown to increase the rate of composite scaffold degradation due to the increase in hydrophilicity.^12^ Thus, the degradation of composite SMP scaffolds (0, 5, and 10 wt % BG) was evaluated. Degradation rates increased with increasing BG levels when tested under accelerated conditions (0.2 M NaOH, pH = 7.4, 37 °C) observed in terms of gravimetric loss, as well as visual and SEM inspection (Figures 6, S13, S14 and Table S10). It is notable that just 5 and 10 wt % BG produced an appreciable effect. The polymer matrix also impacted degradation rates. Faster degradation rates were observed among composite scaffolds based on *star*-PCL (∼28–35%) versus *linear*-PCL (∼32–42%) due to lower PCL % crystallinity. Semi-IPN composite scaffolds degraded significantly faster than the corresponding non-semi-IPN scaffold, attributed to not only the aforementioned phase separation effect, but also the loss of PLLA crystallinity. Overall, composite scaffolds SS-5% and SS-10% exhibited the fastest rates of degradation. Under non-accelerated conditions (1× PBS, pH = 7.4, 37 °C), composite scaffolds, particularly semi-IPNs, exhibited faster degradation rates as well (Figures S15 and S16 and Table S11).

### 3.6 Bioactivity

**Figure 7:**
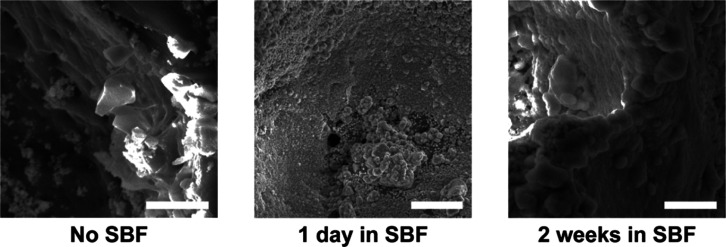
SEM images of HAp growth on a L-5% scaffold before SBF exposure, and after 1 day and 2 weeks in SBF [1×] (scale bars = 20 μm).

Bioactivity was assessed by exposing scaffolds (0, 5 and 10% BG) to SBF (1×). Prior to submersion, SEM images revealed the presence of BG on composite scaffold pore walls that was absent for non-BG scaffolds (Figures S4 and S17). Evidence of HAp mineralization was assessed via SEM after 1 day (Figure S18) and 2 weeks (Figure S19) of submersion in SBF (1×). The BG-free scaffolds showed no signs of mineralization, while all composite scaffolds formed a layer of HAp on pore walls at just 1 day and became more pronounced at 2 weeks (Figure 7). To confirm the identity of the mineralization as that of HAp, EDS was utilized to determine the Ca/P ratios as they are unique to BG (Ca/P ratio = 5)^17^ and HAp (Ca/P ratio = 1.67)^40^ (Figure 2 and Figure S20a–d). For composite scaffolds, there was an increase in the Ca/P molar ratio from the 1 day time-point (Ca/P = 1.494) to the 2 week time-point (Ca/P = 1.677) (Figure S20c,d). Thus, it appears that HAp nucleation begins in just 1 day on composite scaffold pore wall surfaces, indicative of potent bioactivity.

## 4 Conclusions

An off-the-shelf scaffold that possesses key functional properties could advance a regenerative engineering approach to heal bone CMF bone defects. Our previously reported “self-fitting” SMP scaffolds based on PCL importantly provided the ability to conformally fit into irregular geometries and to heal bone defects. The requisite heat required for fitting could be reduced for scaffolds based on *star*-PCL-TA (*T*_m_ ∼45 °C) versus *linear*-PCL-DA (*T*_m_ ∼55 °C) for improved tissue safety. Semi-IPN scaffolds incorporating *linear*- or *star*-PLLA (3:1 wt %: PCL/PLLA) increased degradations rates (due to phase separation effects) and, in some cases, increased modulus. Despite their useful and tunable properties, these SMP scaffolds lack innate bioactivity, and additional increases in degradation rates are predicted to be beneficial. Thus, BG (45S5 BG) was incorporated at varying levels (up to 30 wt %) to form composite scaffolds based on two PCL-only systems prepared with the *linear*- and *star*-PCL macromers (L, S), and four semi-IPN systems based on combining with *linear*- and *star*-PLLA (LL, LS, SL, SS). The SCPL process was not amenable to combining the BG into the macromer solution due to BG settling, so modifications to fabrication were made. Termed the “glass-in-salt mold” method, the BG was combined with the fused salt template, and the macromer solution then casted. This produced composite scaffolds with targeted BG levels, and effective cross-linking of PCL macromers was retained. SEM images revealed that the BG was concentrated on the pore wall surfaces rather than pore struts. This distribution of BG was associated with many of the observed properties of the composite scaffolds. This includes a lack of brittleness for all compositions. At 20 and 30 wt % BG, the integrity of the pore walls was diminished, leading to reduced *E* and CS values. However, composite scaffolds containing 2.5, 5, or 10 wt % BG generally produced enhancements in these properties over non-BG controls. The presence of BG did not lead to appreciable changes of PCL % crystallinity, contributing to robust mechanical properties and excellent shape memory behavior. PLLA % crystallinity was notably lost for all semi-IPN composite scaffolds, perhaps due to its greater interaction with the BG. Still, this did not have a notable impact on mechanical properties. Composite scaffolds with 5 and 10 wt % BG degraded faster versus non-BG scaffolds, owing to the enhanced hydrophilicity of the pore walls. For semi-IPN composite scaffolds, the loss of PLLA crystallinity was a likely additional contributor to the enhanced rates of degradation. Composite scaffolds with 5 and 10 wt % BG were also bioactive. After exposure to SBF (1×), SEM/EDS analysis revealed that HAp began to cover the pore walls after just 1 day and was present at greater levels at 2 weeks. Overall, the formation of BG-containing SMP composite scaffolds using the “glass-in-salt mold” method allowed the localization of BG on the pore walls. Thus, at even low BG levels (≤10 wt %), substantial and favorable changes could be made to bioactivity and degradation, without producing brittleness or a loss of shape memory behavior. The favorable material properties of these bioactive, “self-fitting” BG composite scaffolds are predicted to afford their utility to heal complex bone defects. To confirm this, future studies of these scaffolds will include *in vitro* cell culture (e.g., support of human MSC osteogenesis and induction of mineralization), and *in vivo* analyses in appropriate bone defect models (e.g., rat or rabbit calvarial defects) as described in our prior reports.

## Supporting Information Available

Scaffold fabrication methods; scaffold compositions; TGA data; SEM images; sol content data; pore morphology; Alizarin Red S staining data; pore interconnectivity, size, and porosity data; compressive mechanical properties; DSC data; shape memory photos and data; accelerated degradation data and SEM images; nonaccelerated degradation data and SEM images; SBF testing-SEM/EDS images and data (PDF)

## Nomenclature

CMF: craniomaxillofacial
HAp: hydroxyapatite
MSC: mesenchymal stem cell
BG: 45S5 bioglass
SMP: shape memory polymer
PCL: poly(ε-caprolactone)
SCPL: solvent-cast particulate leaching
*T*_m_: melt transition temperature
Semi-IPN: semi-interpenetrating network
PLLA: poly(l-lactic acid)
PDA: polydopamine
SBF: simulated body fluid
PDA: polydopamine
PDMS: polydimethylsiloxane
PMHS: polymethylhydrosiloxane
*T*_g_: glass transition temperature
TGA: thermal gravimetric analysis
SEM: scanning electron microscopy
rb: round bottom
ON: overnight
RT: room temperature
*E*: compressive modulus
CS: compressive strength
DSC: differential scanning calorimetry
*R*_f_: shape fixity
*R*_r_: shape recovery
EDS: energy-dispersive X-ray spectroscopy
