# Supplementary material for: Shape Memory Polymer Bioglass Composite Scaffolds Designed to Heal Complex Bone Defects
Source: ACS Biomater Sci Eng. 2024 Oct 4;10(10):6509–19. doi: 10.1021/acsbiomaterials.4c01073 (PMC11480937; doi:10.1021/acsbiomaterials.4c01073)
Supplement: Supplementary file 1 — ab4c01073_si_001.pdf [file ab4c01073_si_001.pdf]

Supporting Information

Shape Memory Polymer (SMP) Bioglass Composite Scaffolds Designed to Heal Complex Bone Defects

*Brandon M. Nitschke,[1] Elizabeth A. Butchko,[1] MaryGrace N. Wahby,[1] Kaylee M. Breining,[2] Alexander E. Konz,[1] and Melissa A. Grunlan[1,2,3]**

[1]Department of Biomedical Engineering, Texas A&M University, College Station, Texas 77843, United States.

[2]Department of Materials Science and Engineering, Texas A&M University, College Station, Texas 77843, United States.

[3]Department of Chemistry, Texas A&M University, College Station, Texas 77843, United States.

*Corresponding author email: mgrunlan@tamu.edu

30 Pages

20 Figures

11 Tables

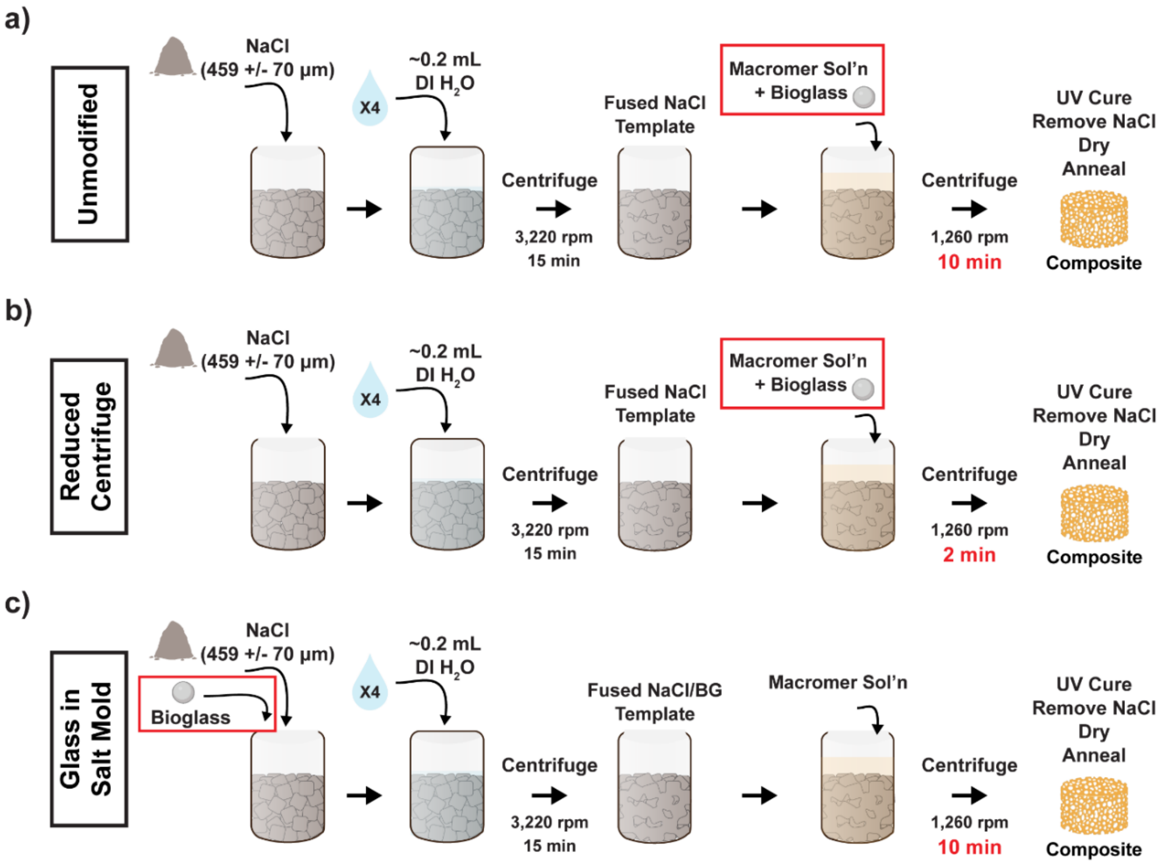

Figure S1. Potential SCPL fabrication techniques to prepare composite SMP scaffolds: **(a)** "Unmodified" (i.e., original SCPL protocol used previously to prepare BG-free SMP scaffolds), **(b)** "reduced centrifuge", and **(c)** "glass-in-salt mold." Method "**c**" was selected to prepare composite scaffolds reported herein.

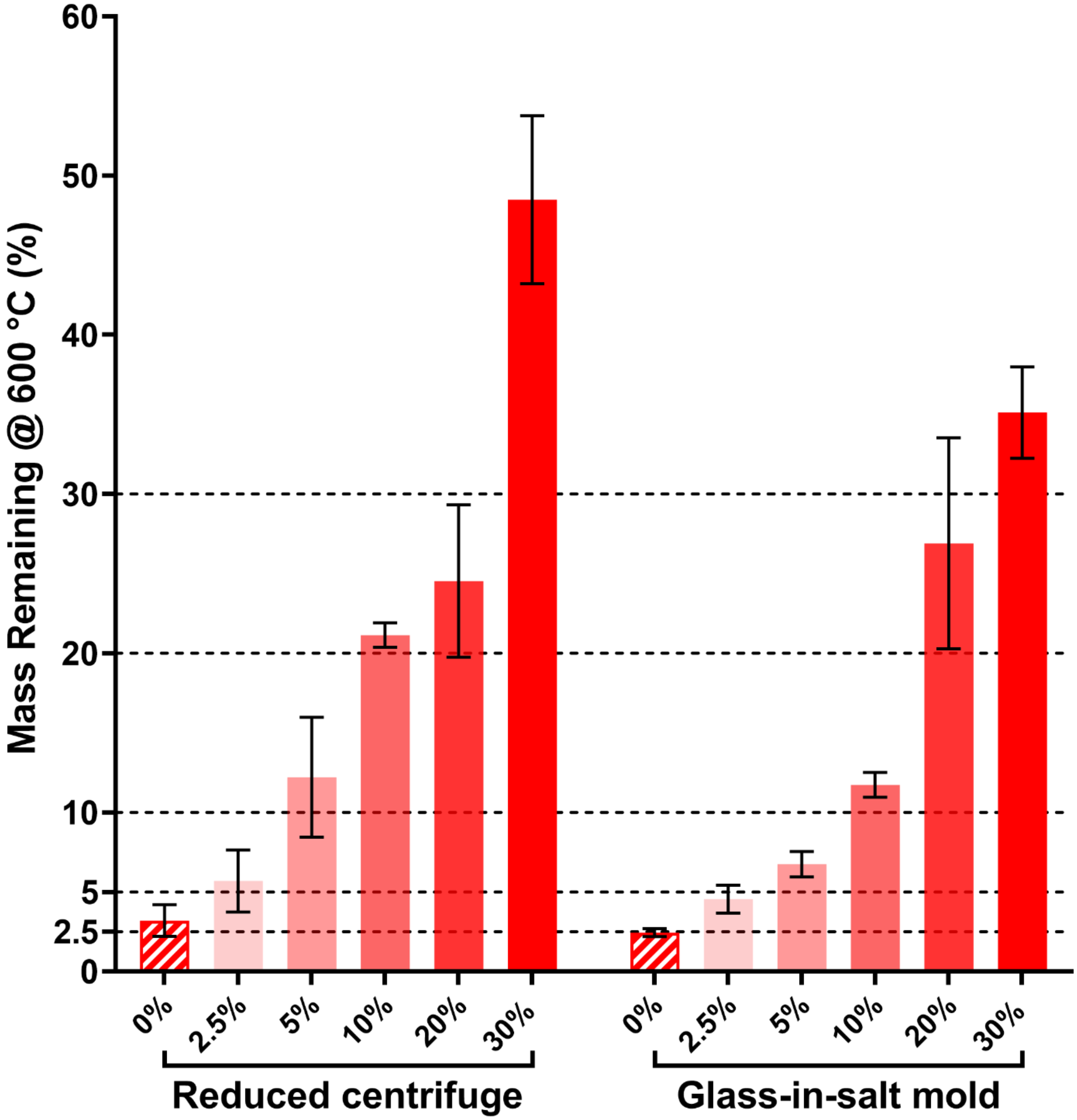

Figure S2. TGA plateau values for the "reduced centrifuge" (per Fig. S1b) and "glass-in-salt mold" (per Fig. S1c) SCPL fabrication techniques. The "glass-in-salt mold" method produced composite scaffolds with BG wt% values closer to expected values, and thus was used to prepare composite SMP scaffolds evaluated herein.

Table S1. TGA plateau values (600 °C for the "glass-in-salt mold" and "reduced centrifuge" SCPL fabrication techniques).

Scaffold	"Reduced centrifuge" mass remaining (%)	"Glass-in-salt mold" mass remaining (%)
L-0%	3.20 ± 0.99	2.44 ± 0.25
L-2.5%	5.69 ± 1.94	4.55 ± 0.87
L-5%	12.20 ± 3.76	6.74 ± 0.80
L-10%	21.13 ± 0.77	11.73 ± 0.78
L-20%	24.53 ± 4.78	26.89 ± 6.63
L-30%	48.48 ± 5.28	35.11 ± 2.88

Table S2. Scaffold compositions.

Notation	Bioglass	Macromers: *Linear*-PCL-DA or *Star*-PCL-TA		Macromers: *Linear*-PLLA or *Star*-PLLA	
	wt%	Architecture	wt%	Architecture	wt%
Non-Semi-IPNs					
L-0%	0%		100%	-	-
L-2.5%	2.5%		97.5%	-	-
L-5%	5%	*Linear*	95%	-	-
L-10%	10%		90%	-	-
L-20%	20%		80%	-	-
L-30%	30%		70%	-	-
S-0%	0%		100%	-	-
S-2.5%	2.5%		97.5%	-	-
S-5%	5%	*Star*	95%	-	-
S-10%	10%		90%	-	-
S-20%	20%		80%	-	-
S-30%	30%		70%	-	-
Semi-IPNs					
LL-0%	0%		75%		25%
LL-2.5%	2.5%		73.125%		24.375%
LL-5%	5%	*Linear*	71.25%	*Linear*	23.75%
LL-10%	10%		67.5%		22.5%
LL-20%	20%		60%		20%
LL-30%	30%		52.5%		17.5%
LS-0%	0%		75%		25%
LS-2.5%	2.5%		73.125%		24.375%
LS-5%	5%	*Linear*	71.25%	*Star*	23.75%
LS-10%	10%		67.5%		22.5%
LS-20%	20%		60%		20%
LS-30%	30%		52.5%		17.5%
SL-0%	0%		75%		25%
SL-2.5%	2.5%		73.125%		24.375%
SL-5%	5%	*Star*	71.25%	*Linear*	23.75%
SL-10%	10%		67.5%		22.5%
SL-20%	20%		60%		20%
SL-30%	30%		52.5%		17.5%
SS-0%	0%		75%		25%
SS-2.5%	2.5%		73.125%		24.375%
SS-5%	5%	*Star*	71.25%	*Star*	23.75%
SS-10%	10%		67.5%		22.5%
SS-20%	20%		60%		20%
SS-30%	30%		52.5%		17.5%

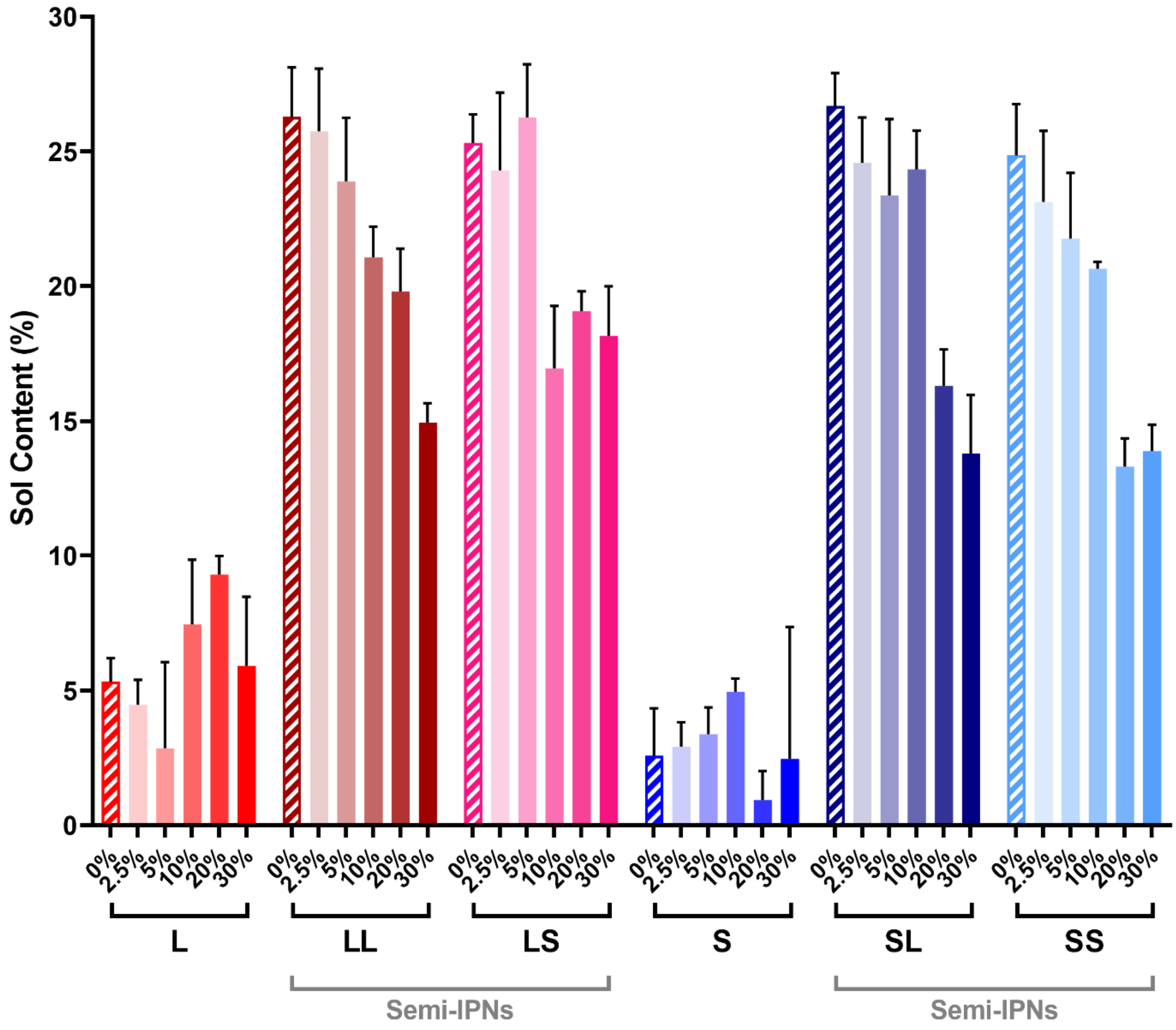

Figure S3. Sol content values of scaffolds.

Table S3. Sol content (%) of scaffolds

Scaffold	Sol content (%)
L-0%	5.33 ± 0.88
L-2.5%	4.47 ± 0.93
L-5%	2.86 ± 3.20
L-10%	7.45 ± 2.40
L-20%	9.30 ± 0.68
L-30%	5.91 ± 2.57
LL-0%	26.27 ± 1.84
LL-2.5%	25.74 ± 2.33
LL-5%	23.89 ± 2.37
LL-10%	21.07 ± 1.14
LL-20%	19.80 ± 1.59
LL-30%	14.94 ± 0.72
LS-0%	25.32 ± 1.05
LS-2.5%	24.30 ± 2.89
LS-5%	26.26 ± 1.97
LS-10%	16.95 ± 2.31
LS-20%	19.07 ± 0.74
LS-30%	18.16 ± 1.84
S-0%	2.58 ± 1.76
S-2.5%	2.92 ± 0.91
S-5%	3.37 ± 1.00
S-10%	4.96 ± 0.49
S-20%	0.93 ± 1.08
S-30%	2.46 ± 4.91
SL-0%	26.69 ± 1.22
SL-2.5%	24.57 ± 1.69
SL-5%	23.37 ± 2.83
SL-10%	24.34 ± 1.44
SL-20%	16.31 ± 1.35
SL-30%	13.80 ± 2.17
SS-0%	24.85 ± 1.91
SS-2.5%	23.13 ± 2.63
SS-5%	21.77 ± 2.44
SS-10%	20.65 ± 0.25
SS-20%	13.30 ± 1.05
SS-30%	13.88 ± 0.99

Table S4. TGA wt% plateau values at 600 °C.

Scaffold	Wt% remaining at 600 °C
L-0%	2.44 ± 0.25
L-2.5%	4.55 ± 0.87
L-5%	6.74 ± 0.80
L-10%	11.73 ± 0.78
L-20%	26.89 ± 6.63
L-30%	35.11 ± 2.88
LL-0%	3.15 ± 0.29
LL-2.5%	3.92 ± 0.53
LL-5%	9.35 ± 2.89
LL-10%	9.28 ± 2.20
LL-20%	18.78 ± 1.78
LL-30%	31.43 ± 7.13
LS-0%	3.15 ± 0.92
LS-2.5%	3.62 ± 0.69
LS-5%	9.43 ± 3.02
LS-10%	14.05 ± 1.50
LS-20%	20.54 ± 2.08
LS-30%	29.19 ± 3.21
S-0%	4.77 ± 2.72
S-2.5%	5.40 ± 0.54
S-5%	6.99 ± 0.73
S-10%	12.88 ± 2.69
S-20%	20.04 ± 2.31
S-30%	35.74 ± 0.99
SL-0%	3.40 ± 1.04
SL-2.5%	4.94 ± 1.02
SL-5%	5.68 ± 1.05
SL-10%	11.48 ± 3.45
SL-20%	20.66 ± 4.54
SL-30%	31.94 ± 3.33
SS-0%	2.80 ± 0.65
SS-2.5%	5.16 ± 0.64
SS-5%	7.55 ± 0.99
SS-10%	12.34 ± 2.24
SS-20%	20.31 ± 1.19
SS-30%	31.97 ± 2.01

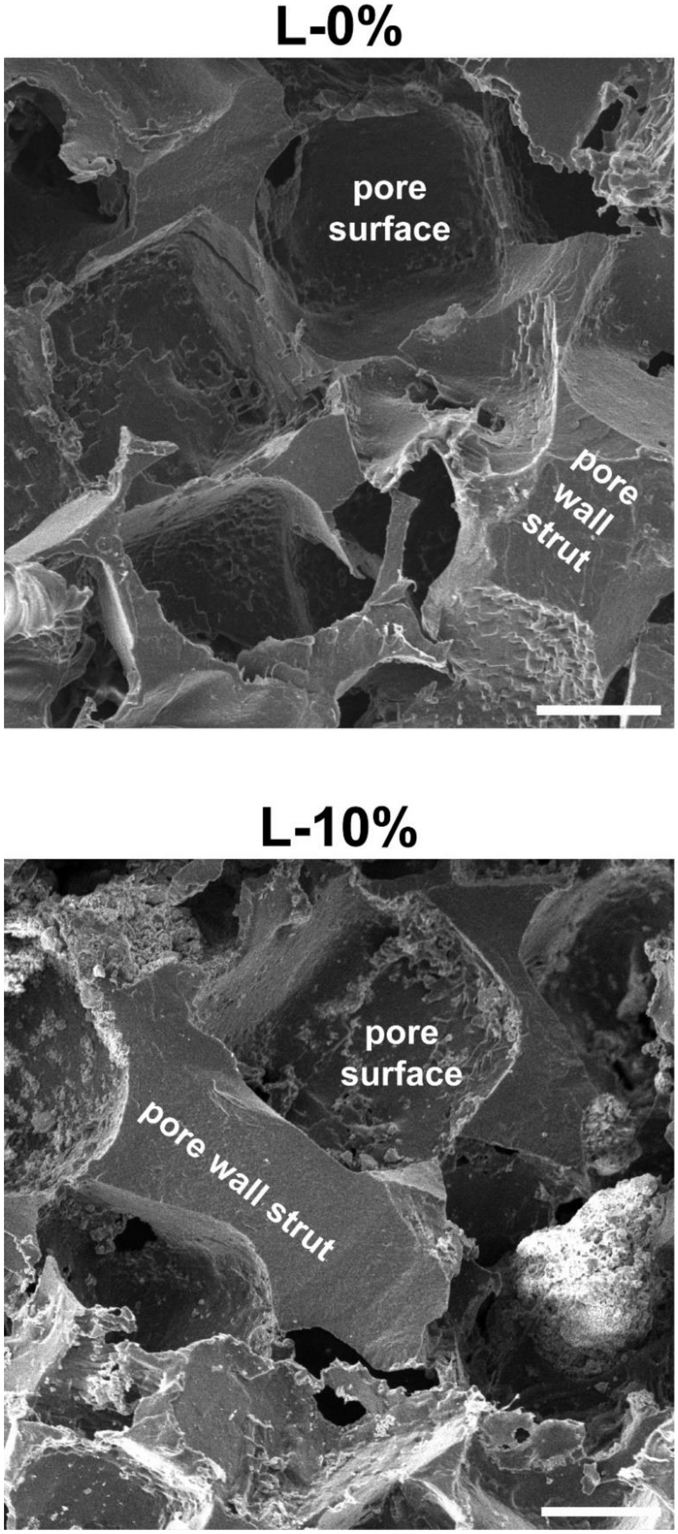

Figure S4. SEM cross-sections of L-0% and L-10% scaffolds. BG was revealed to be concentrated on the pore wall surfaces rather than within the pore wall strut, attributed to the “glass-in-salt mold” method (scale bars = 100 μm).

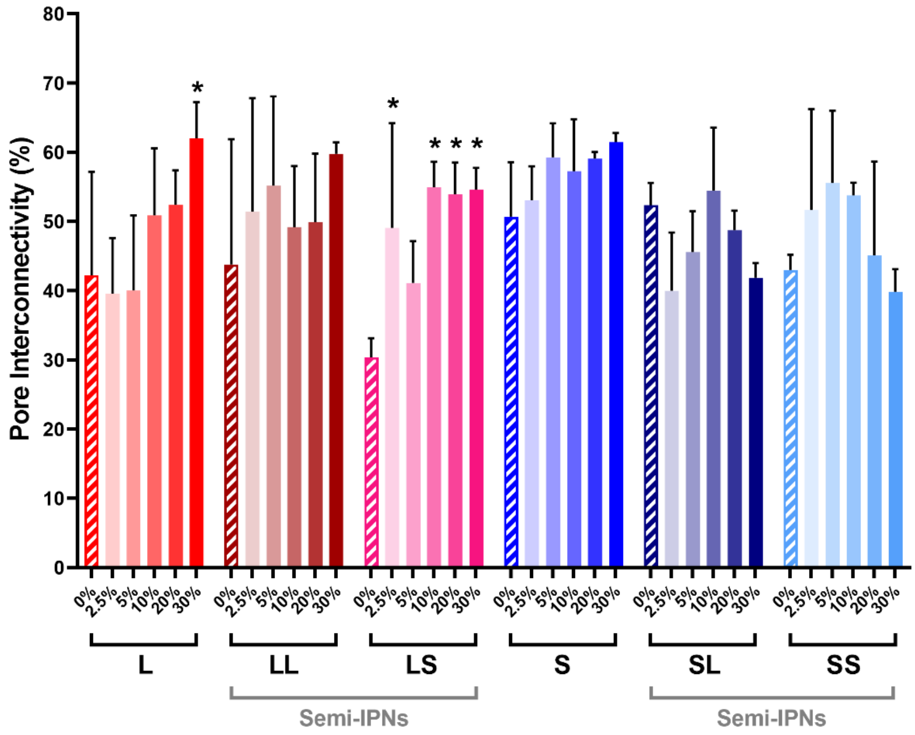

Figure S5. Pore interconnectivity of scaffolds; *$p < 0.05$ vs. 0% BG of analogous macromer composition.

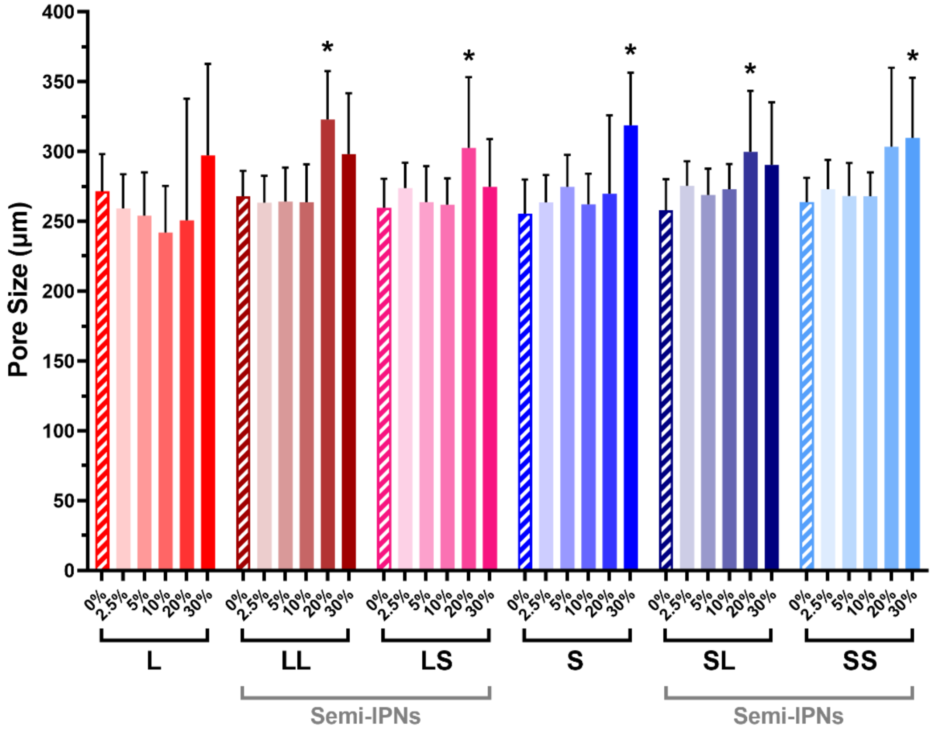

Figure S6. Pore size of scaffolds; *$p < 0.05$ vs. 0% BG of analogous macromer composition.

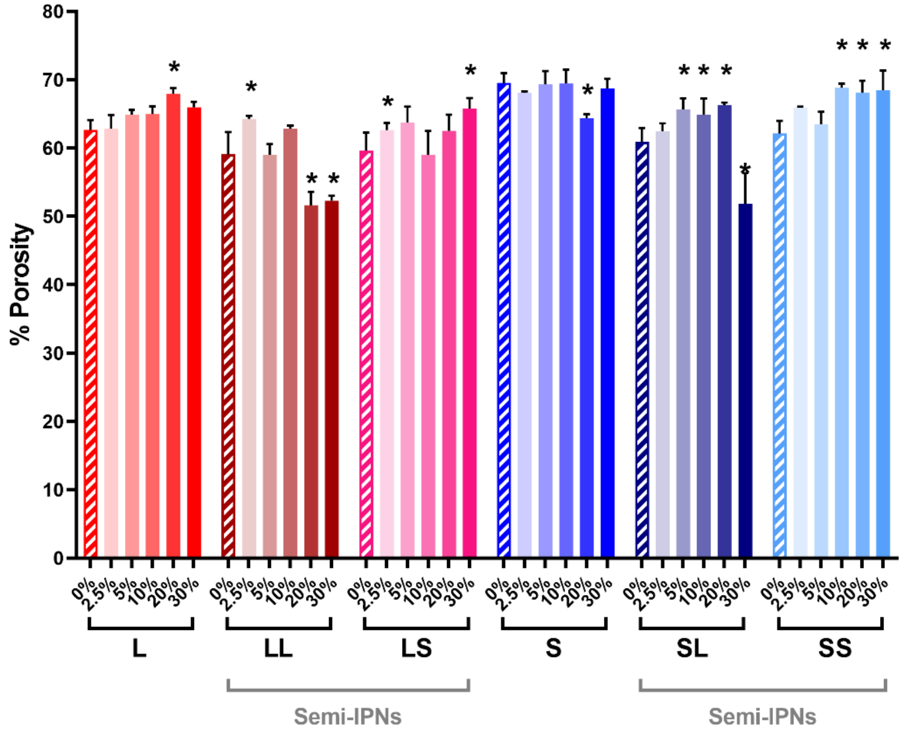

Figure S7. Percent porosity of scaffolds *$p < 0.05$ vs. 0% BG of analogous macromer composition.

Table S5. Porous properties of scaffolds.

Scaffold	Pore Interconnectivity (%)	Pore Size (µm)	Porosity (%)
L-0%	42.24 ± 14.92	271.31 ± 26.74	62.65 ± 1.45
L-2.5%	39.57 ± 8.03	259.17 ± 24.40	62.82 ± 2.01
L-5%	40.04 ±10.85	253.98 ± 30.90	64.91 ± 0.67
L-10%	50.89 ± 9.67	241.80 ± 33.48	64.97 ± 1.13
L-20%	52.40 ± 4.97	250.64 ± 87.06	67.92 ± 0.84
L-30%	62.02 ± 5.22	297.02 ± 65.71	65.96 ± 0.81
LL-0%	43.79 ± 18.10	267.84 ± 18.15	59.07 ± 3.26
LL-2.5%	51.44 ± 16.37	263.27 ± 19.22	64.28 ± 0.42
LL-5%	55.18 ± 12.89	264.03 ± 24.42	58.99 ± 1.60
LL-10%	49.19 ± 8.82	263.52 ± 27.21	62.81 ± 0.47
LL-20%	49.88 ± 9.93	322.79 ± 34.65	51.65 ± 1.95
LL-30%	59.78 ± 1.65	298.06 ± 43.53	52.30 ± 0.72
LS-0%	30.37 ± 2.74	259.69 ± 20.69	59.62 ± 2.65
LS-2.5%	49.08 ± 15.13	273.78 18.08	62.64 ± 1.04
LS-5%	41.15 ± 6.01	263.73 ± 25.64	63.72 ± 2.34
LS-10%	54.93 ± 3.69	261.87 ± 18.75	59.01 ± 3.51
LS-20%	53.92 ± 4.59	302.49 ± 50.62	62.51 ± 2.38
LS-30%	54.62 ± 3.11	274.58 ± 34.22	65.81 ± 1.51
S-0%	50.67 ± 7.88	255.49 ± 24.35	69.51 ± 1.45
S-2.5%	53.05 ± 4.92	263.38 ± 19.71	68.08 ± 0.23
S-5%	59.27 ± 4.92	274.56 ± 22.95	69.32 ± 1.94
S-10%	57.27 ± 7.49	262.06 ± 22.06	69.46 ± 2.00
S-20%	59.07 ± 0.97	269.72 ± 56.09	64.38 ± 0.58
S-30%	61.47 ± 1.33	318.61 ± 37.74	68.71 ± 1.42
SL-0%	52.36 ± 3.20	257.85 ± 22.23	60.87 ± 2.05
SL-2.5%	40.02 ± 8.38	275.29 ± 17.60	62.46 ± 1.15
SL-5%	45.58 ± 5.89	268.74 ± 18.78	65.65 ± 1.64
SL-10%	54.47 ± 9.08	272.83 ± 18.11	64.90 ± 2.36
SL-20%	48.72 ± 2.84	299.63 ± 43.77	66.28 ± 0.37
SL-30%	41.88 ± 2.11	290.23 ± 44.91	51.88 ± 4.44
SS-0%	42.99 ± 2.20	263.79 ± 17.24	62.11 ± 1.88
SS-2.5%	51.68 ± 14.57	273.03 ± 20.85	65.88 ± 0.20
SS-5%	55.55 ± 10.46	268.15 ± 23.58	63.50 ± 1.82
SS-10%	53.75 ± 1.85	267.84 ± 16.99	68.82 ± 0.62
SS-20%	45.09 ± 13.56	303.23 ± 56.66	68.12 ± 1.74
SS-30%	39.83 ± 3.29	309.62 ± 43.20	68.46 ± 2.90

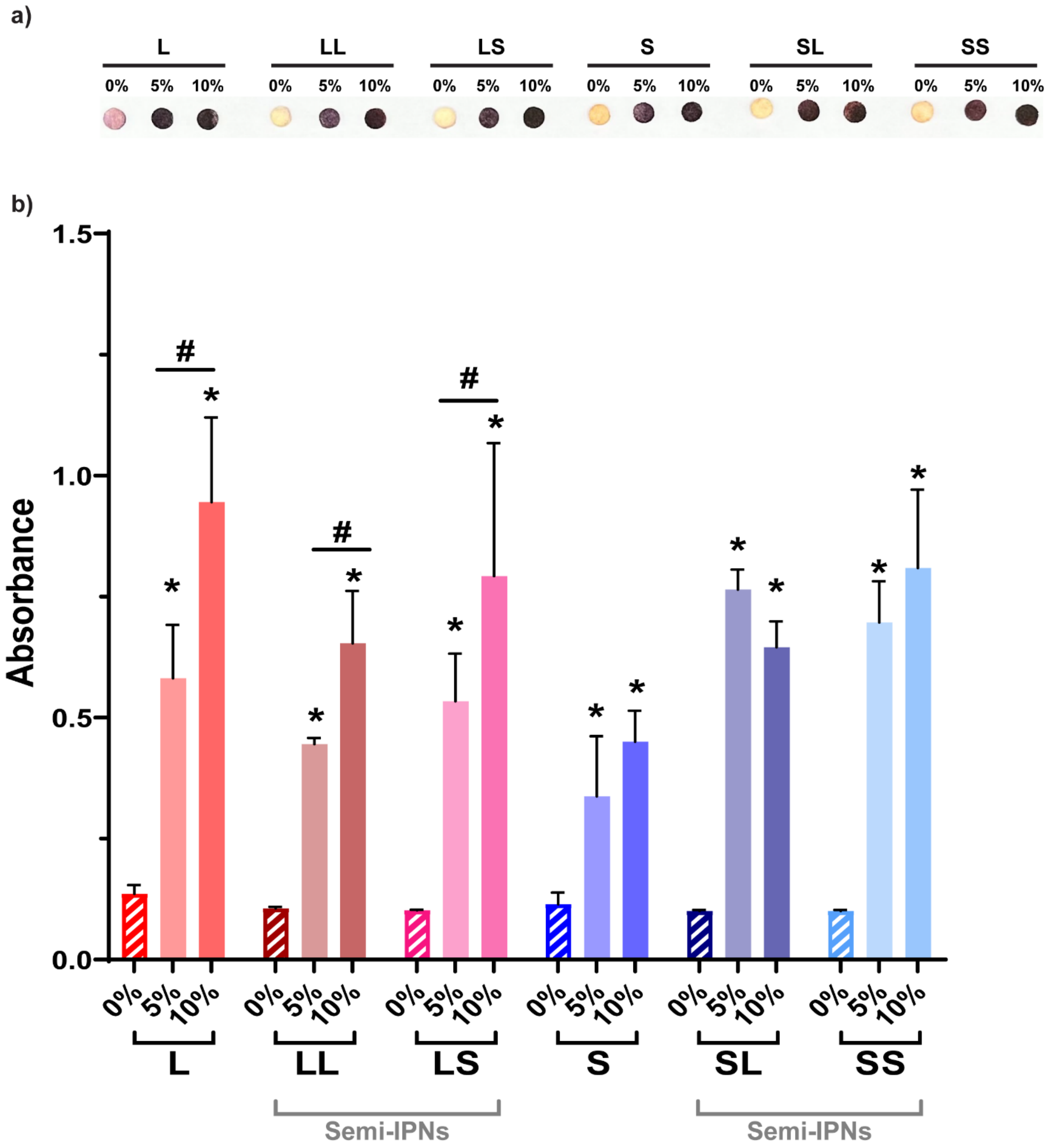

Figure S8. Alizarin Red S staining of "as is" scaffolds (i.e., prior to exposure to SBF). **(a)** Photos of stained scaffolds. **(b)** Absorbances of stained scaffolds; *$p < 0.05$ vs. 0% BG of analogous macromer composition; #$p < 0.05$ compared between 5% and 10% BG of analogous macromer composition.

Table S6. Absorbance values of scaffolds stained with Alizarin Red S.

Scaffold	Absorbance
L-0%	0.14 ± 0.02
L-5%	0.58 ± 0.11
L-10%	0.95 ± 0.18
LL-0%	0.11 ± 0.00
LL-5%	0.45 ± 0.01
LL-10%	0.65 ± 0.11
LS-0%	0.10 ± 0.00
LS-5%	0.53 ± 0.10
LS-10%	0.79 ± 0.27
S-0%	0.11 ± 0.02
S-5%	0.34 ± 0.12
S-10%	0.45 ± 0.06
SL-0%	0.10 ± 0.00
SL-5%	0.76 ± 0.04
SL-10%	0.65 ± 0.05
SS-0%	0.10 ± 0.00
SS-5%	0.70 ± 0.09
SS-10%	0.81 ± 0.16

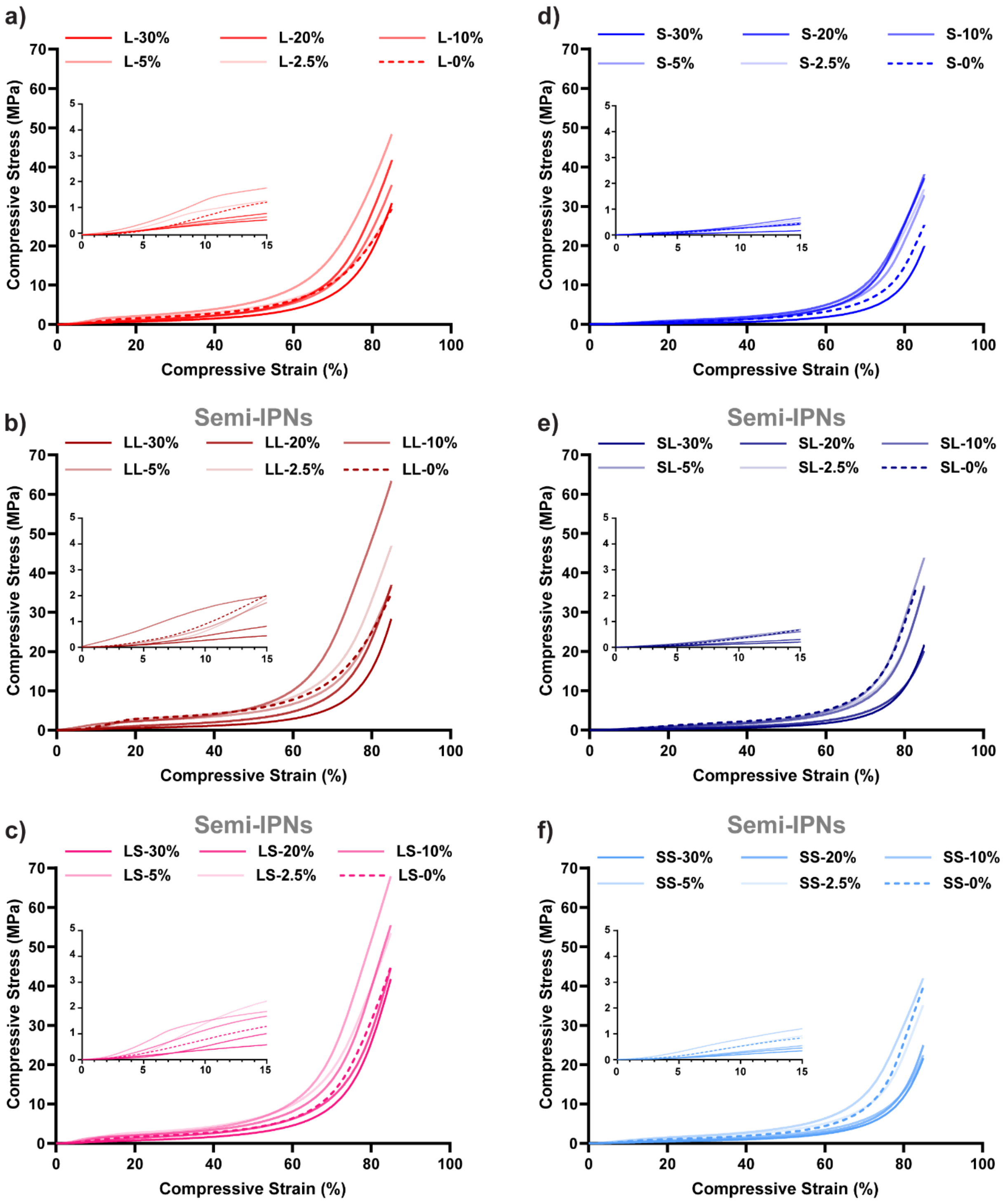

Figure S9. Representative compressive stress-strain curves of **(a)** L, **(b)** LL, **(c)** LS, **(d)** S, **(e)** SL, and **(f)** SS series scaffolds. Inset graph displays region where modulus was determined (i.e., 0-10% strain).

Table S7. Compressive mechanical properties of scaffolds.

Scaffold	Modulus (MPa)	Strength (MPa)	Toughness (MJ m^{-3})
L-0%	7.38 ± 1.87	31.85 ± 7.23	5.16 ± 0.89
L-2.5%	9.43 ± 1.94	36.93 ± 4.37	5.96 ± 0.65
L-5%	13.30 ± 2.72	50.38 ± 4.19	8.09 ± 0.68
L-10%	6.04 ± 2.31	39.19 ± 4.72	5.55 ± 0.89
L-20%	5.79 ± 1.06	41.94 ± 3.50	5.98 ± 0.71
L-30%	4.63 ± 1.07	36.34 ± 5.52	4.50 ± 0.78
LL-0%	10.78 ± 3.33	38.95 ± 3.94	6.72 ± 0.54
LL-2.5%	7.55 ± 2.98	44.67 ± 10.09	6.86 ± 1.48
LL-5%	9.47 ± 3.37	38.27 ± 8.57	5.79 ± 0.98
LL-10%	15.35 ± 3.92	57.49 ± 5.21	8.54± 1.34
LL-20%	4.44 ± 0.96	38.17 ± 6.24	4.29± 0.59
LL-30%	3.11 ± 0.29	35.40 ± 6.25	3.65± 0.65
LS-0%	7.17 ± 2.12	43.70 ± 2.91	6.20 ± 0.26
LS-2.5%	13.50 ± 4.91	51.17 ± 8.86	8.22 ± 1.88
LS-5%	14.04 ± 4.21	56.08 ± 8.80	8.55 ±1.58
LS-10%	11.96 ± 1.61	54.33 ± 5.72	8.26 ± 1.07
LS-20%	6.21 ± 1.12	45.06 ± 2.73	5.70 ± 0.29
LS-30%	3.18 ± 0.60	37.99 ± 3.97	3.97 ± 0.60
S-0%	2.54 ± 0.27	27.88 ± 6.08	3.49 ± 0.36
S-2.5%	2.41 ± 0.48	24.63 ± 6.91	3.22 ± 0.91
S-5%	2.86 ± 0.31	30.38 ± 7.12	3.84 ± 0.74
S-10%	2.69 ± 0.67	33.72 ± 6.44	4.41 ± 0.66
S-20%	2.82 ± 0.50	35.96 ± 2.58	4.24 ± 0.35
S-30%	1.00 ± 0.18	21.37 ± 2.49	2.04 ± 0.23
SL-0%	2.54 ± 0.59	35.69 ± 6.08	4.55 ± 0.72
SL-2.5%	2.76 ± 0.76	35.45 ± 4.77	4.24 ± 0.24
SL-5%	3.58 ± 0.63	40.16 ± 2.98	4.71 ± 0.31
SL-10%	2.68 ± 0.63	36.71 ± 2.03	3.91 ± 0.19
SL-20%	1.77 ± 0.36	20.40 ± 3.35	2.30 ± 0.29
SL-30%	1.54 ± 0.57	20.51 ± 6.87	2.11 ± 0.61
SS-0%	4.27 ± 1.40	38.07 ± 7.85	4.28 ± 1.21
SS-2.5%	5.74 ± 1.50	41.37 ± 9.64	5.15 ± 1.06
SS-5%	6.96 ± 2.89	37.66 ± 9.51	5.47 ± 1.63
SS-10%	2.71 ± 0.89	23.56 ± 5.14	2.81 ± 0.51
SS-20%	2.67 ± 0.49	27.53 ± 3.38	2.97 ± 0.41
SS-30%	2.26 ± 0.43	26.23 ± 3.95	2.82 ± 0.52

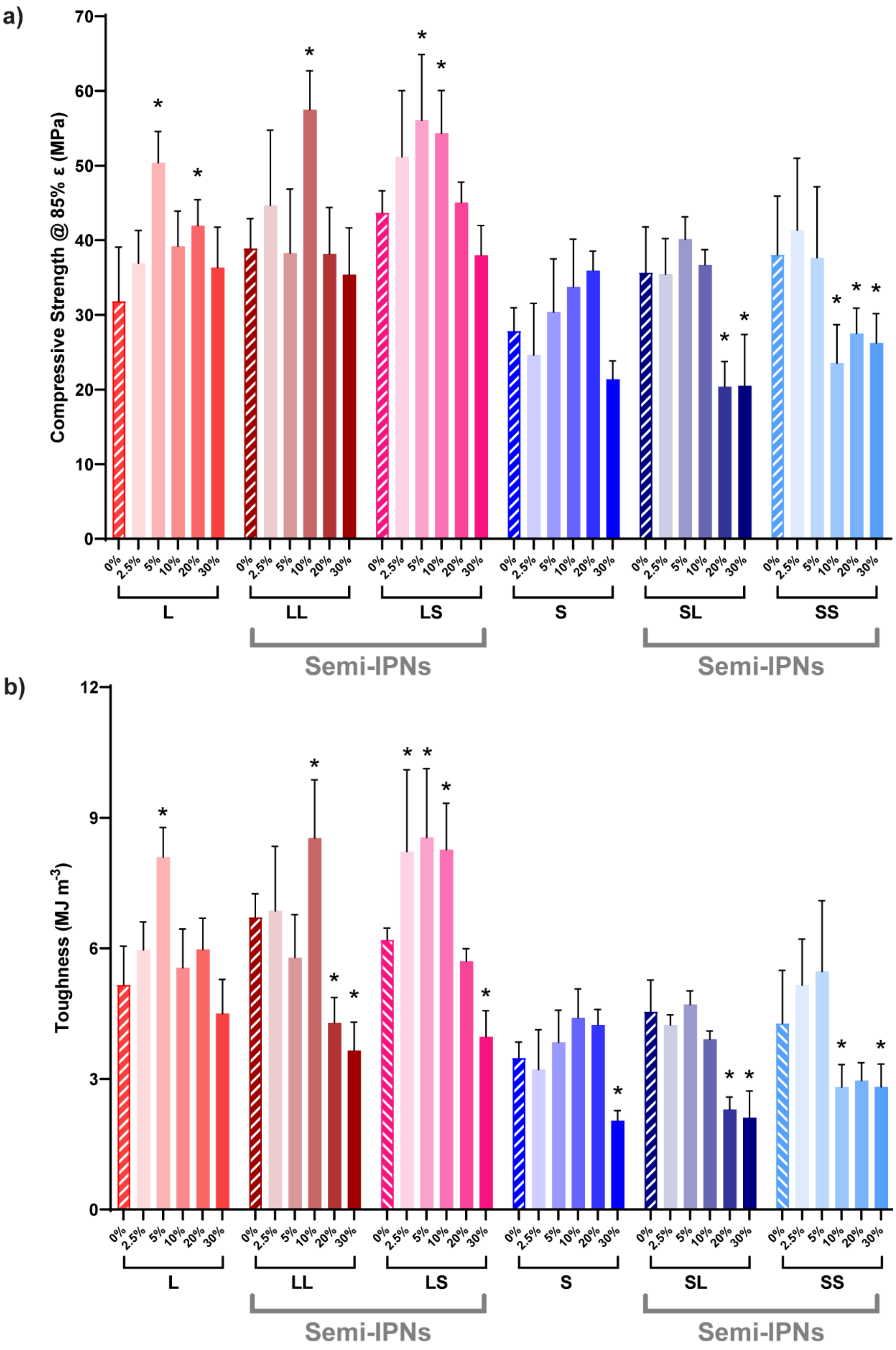

Figure S10. (a) Compressive strength (*CS*), and **(b)** compressive toughness of scaffolds; *$p < 0.05$ vs. 0% BG of analogous macromer composition.

Table S8. Thermal properties of scaffolds.

	PCL		PLLA	
	T_m midpoint (°C)	% crystallinity	T_m midpoint (°C)	% crystallinity
**L-0%**	54.82 ± 0.91	42.20 ± 3.32	-	-
**L-5%**	54.88 ± 0.27	32.80 ± 0.41	-	-
**L-10%**	54.96 ± 0.21	32.09 ± 2.53	-	-
**LL-0%**	53.61 ± 0.06	36.68 ± 0.90	161.80 ± 1.83	47.99 ± 2.69
**LL-5%**	55.44 ± 1.44	37.73 ± 0.48	-	-
**LL-10%**	53.64 ± 0.24	37.70 ± 2.29	-	-
**LS-0%**	55.63 ± 0.80	39.68 ± 2.42	158.19 ± 0.60	41.29 ± 5.67
**LS-5%**	54.60 ± 0.01	41.59 ± 1.20	-	-
**LS-10%**	53.71 ± 0.75	42.28 ± 3.98	-	-
**S-0%**	47.38 ± 0.53	30.50 ± 1.21	-	-
**S-5%**	47.41 ± 0.09	28.03 ± 2.83	-	-
**S-10%**	46.26 ± 0.19	28.96 ± 2.06	-	-
**SL-0%**	48.21 ± 0.79	28.59 ± 1.75	159.32 ± 2.57	43.61 ± 5.41
**SL-5%**	46.65 ± 0.80	27.87 ± 2.14	-	-
**SL-10%**	46.88 ± 0.81	34.56 ± 3.48	-	-
**SS-0%**	46.62 ± 0.18	30.03 ± 0.36	152.86 ± 2.18	29.38 ± 5.38
**SS-5%**	45.77 ± 1.12	32.26 ± 4.27	-	-
**SS-10%**	45.79 ± 0.39	33.47 ± 0.71	-	-

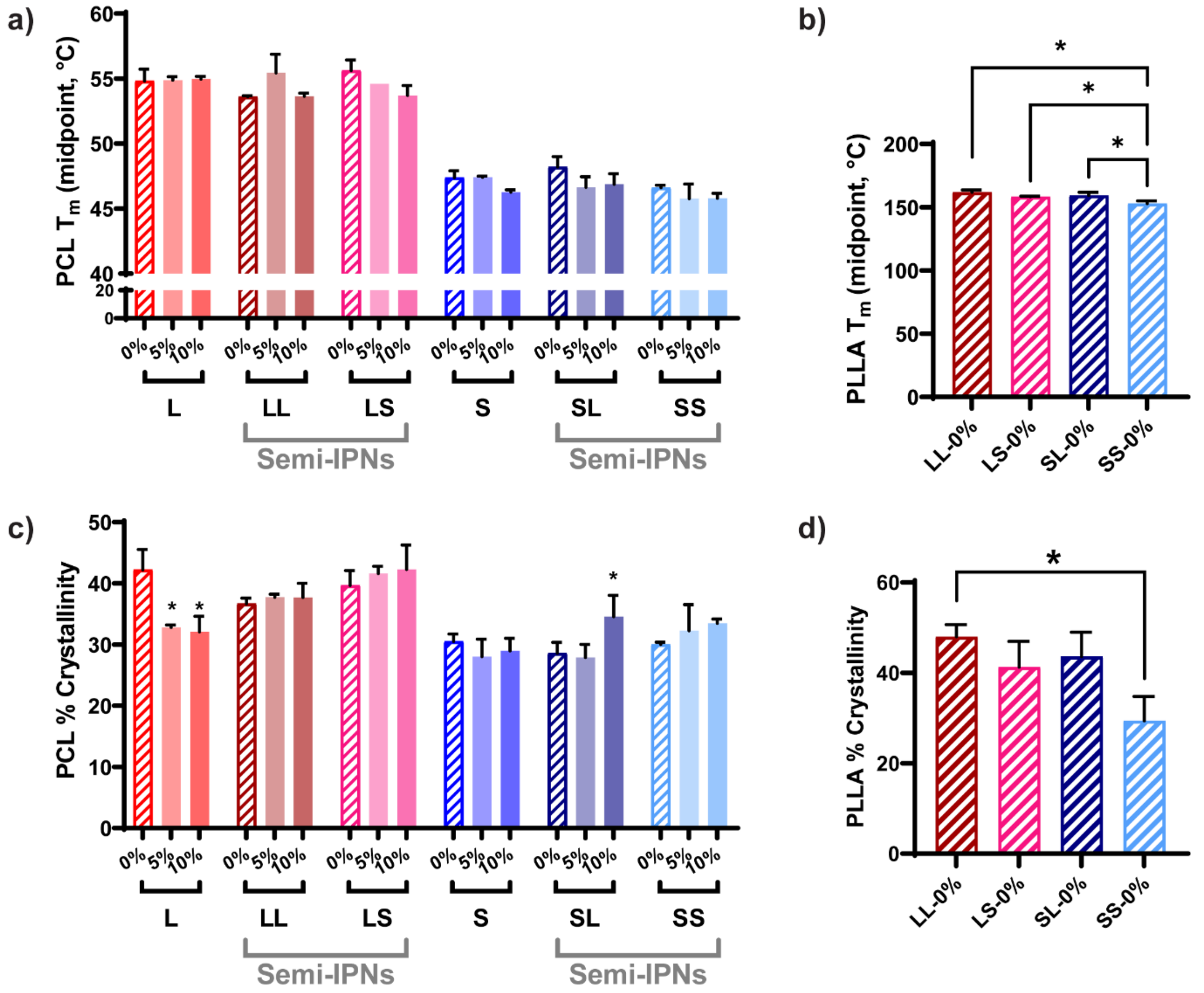

Figure S11. (a) Midpoint T_m of PCL for scaffolds; *$p < 0.05$ vs. 0% BG of analogous macromer composition. **(b)** Midpoint T_m of PLLA for scaffolds; *$p < 0.05$. **(c)** PCL % crystallinity of scaffolds; *$p < 0.05$ vs. 0% BG of analogous macromer composition. **(d)** PLLA % crystallinity of scaffolds; *$p < 0.05$.

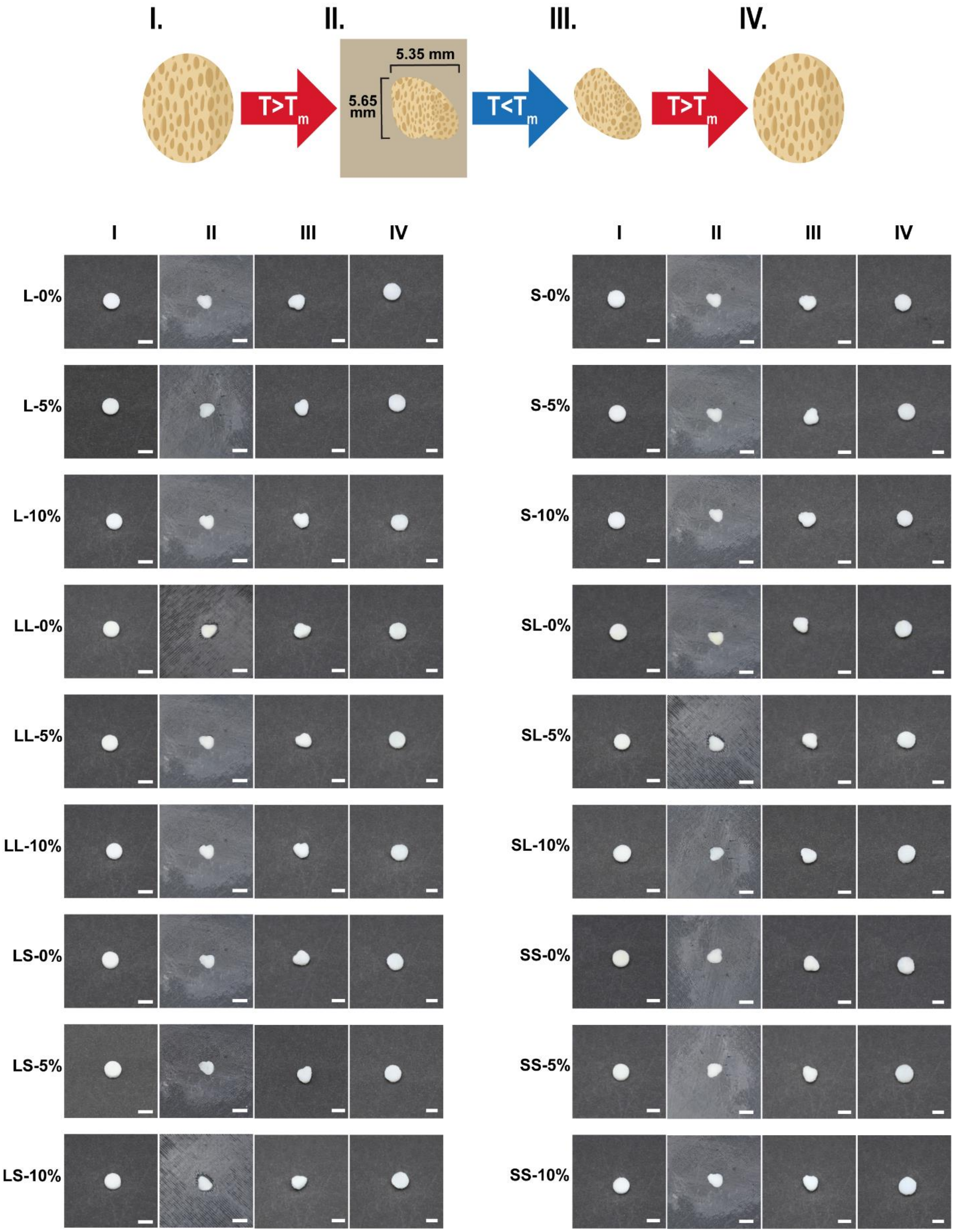

Figure S12. Qualitative shape memory testing displayed high levels of shape fixity and recovery. **[I]** Initial scaffold. **[II]** Scaffold submerged in saline 5 °C higher than its T_m and press-fit into irregular defect. **[III]** Scaffold removed from defect after cooling to RT and maintains its shape (i.e., shape-fixity). **[IV]** Scaffold submerged in warm saline again and recovers to original shape (i.e., shape-recovery) (scale bars = 5 mm).

Table S9. Quantitative shape-memory testing results.

	**Shape Fixity (R_f, %)**	**Shape Recovery (R_r, %)**
**L-0%**	99.93 ± 1.29	98.91 ± 0.81
**L-5%**	101.20 ± 0.70	99.44 ± 0.62
**L-10%**	100.20 ± 1.57	100.90 ± 0.08
**LL-0%**	100.27 ± 0.95	99.74 ± 0.96
**LL-5%**	100.54 ± 1.71	100.31 ± 0.32
**LL-10%**	101.07 ± 0.42	100.66 ± 1.20
**LS-0%**	99.60 ± 0.72	99.35 ± 2.25
**LS-5%**	99.53 ± 1.11	96.98 ± 5.91
**LS-10%**	100.60 ± 1.72	100.44 ± 2.13
**S-0%**	99.67 ± 0.51	100.22 ± 1.10
**S-5%**	99.87 ± 0.51	100.77 ± 2.02
**S-10%**	101.34 ± 0.51	99.98 ± 2.52
**SL-0%**	100.54 ± 1.01	100.98 ± 2.12
**SL-5%**	100.33 ± 0.84	99.90 ± 0.70
**SL-10%**	101.00 ± 0.53	100.21 ± 1.61
**SS-0%**	99.87 ± 0.76	99.34 ± 1.39
**SS-5%**	100.67 ± 1.73	100.81 ± 4.00
**SS-10%**	100.80 ± 0.20	101.22 ± 1.34

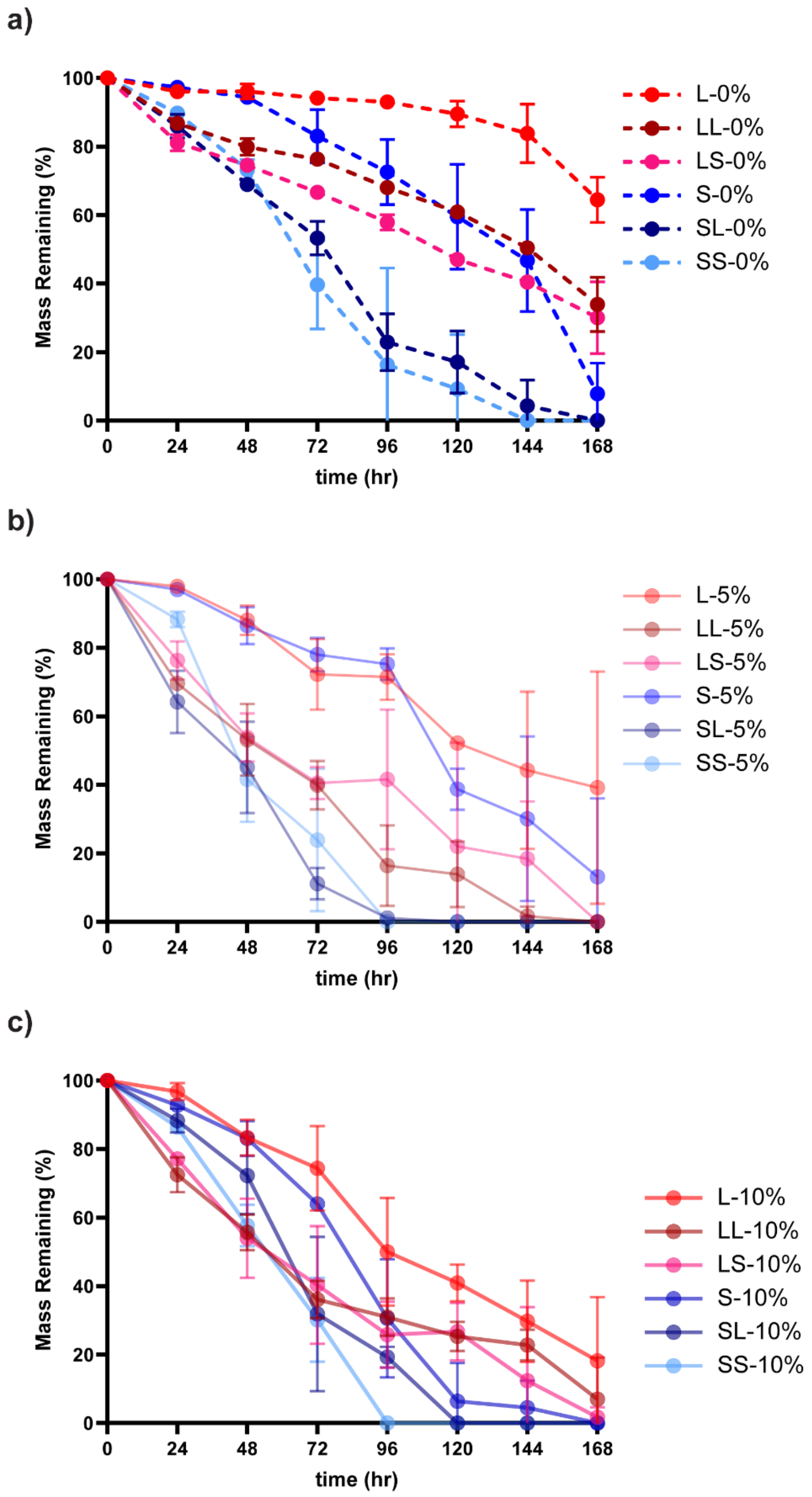

Figure S13. Accelerated degradation (0.2 M NaOH, 37 °C, 60 rpm) of scaffolds grouped by BG wt%: **(a)** 0% BG, **(b)** 5% BG, **(c)** 10%.

Table S10. Accelerated degradation mass remaining (%) of scaffolds over time.

	24 hr	48 hr	72 hr	96 hr	120 hr	144 hr	168 hr
**L-0%**	96.01 ± 1.69	96.08 ± 2.18	94.19 ± 1.20	93.02 ± 1.13	89.49 ± 3.79	83.80 ± 8.56	64.48 ± 6.64
**L-5%**	97.89 ± 0.68	88.05 ± 4.30	72.28 ± 10.32	71.46 ± 6.66	52.20 ± 1.14	44.23 ± 22.95	39.15 ± 33.92
**L-10%**	96.78 ± 2.52	83.30 ± 5.24	74.36 ± 12.37	49.98 ± 15.72	40.94 ± 5.35	29.76 ± 11.87	18.17 ± 18.61
**LL-0%**	86.81 ± 0.21	79.89 ± 2.45	76.33 ± 1.47	68.02 ± 0.17	60.91 ± 1.85	50.45 ± 1.64	33.91 ± 7.93
**LL-5%**	69.58 ± 0.54	53.16 ± 10.47	39.90 ± 7.06	16.41 ± 11.73	13.87 ± 9.57	1.64 ± 2.84	0.00 ± 0.00
**LL-10%**	72.48 ± 5.13	55.70 ± 5.25	36.09 ± 5.45	30.95 ± 5.47	25.29 ± 4.26	22.75 ± 4.50	7.01 ± 12.14
**LS-0%**	81.10 ± 2.35	74.55 ± 1.19	66.67 ± 2.12	57.86 ± 2.27	47.06 ± 1.71	40.41 ± 0.72	30.03 ± 10.45
**LS-5%**	76.29 ± 5.60	53.80 ± 7.01	40.53 ± 4.69	41.58 ± 20.42	22.07 ± 30.27	18.45 ± 16.80	0.00 ± 0.00
**LS-10%**	77.13 ± 1.75	53.97 ± 11.60	40.30 ± 17.21	25.77 ± 9.66	26.72 ± 8.44	12.39 ± 21.45	1.70 ± 2.94
**S-0%**	97.24 ± 0.69	94.41 ± 0.93	83.05 ± 7.73	72.55 ± 9.50	59.52 ± 15.35	46.71 ± 14.90	7.82 ± 8.94
**S-5%**	97.01 ± 1.87	86.51 ± 5.40	78.04 ± 4.92	75.22 ± 4.60	38.75 ± 5.98	30.12 ± 24.03	13.18 ± 22.83
**S-10%**	92.78 ± 1.43	83.08 ± 5.09	63.95 ± 1.49	30.56 ± 17.24	6.41 ± 11.10	4.53 ± 7.84	0.00 ± 0.00
**SL-0%**	85.95 ± 3.49	68.95 ± 1.83	53.30 ± 4.91	22.88 ± 8.31	17.08 ± 9.09	4.32 ± 7.49	0.00 ± 0.00
**SL-5%**	64.21 ± 9.05	45.07 ± 13.31	11.15 ± 4.62	1.10 ± 1.90	0.00 ± 0.00	0.00 ± 0.00	0.00 ± 0.00
**SL-10%**	88.28 ± 3.44	72.25 ± 11.24	31.88 ± 22.56	19.26 ± 3.06	0.00 ± 0.00	0.00 ± 0.00	0.00 ± 0.00
**SS-0%**	89.75 ± 1.33	73.15 ± 3.02	39.65 ± 12.89	16.32 ± 28.26	9.20 15.94	0.00 ± 0.00	0.00 ± 0.00
**SS-5%**	88.27 ± 2.25	41.62 ± 12.44	23.85 ± 20.78	0.00 ± 0.00	0.00 ± 0.00	0.00 ± 0.00	0.00 ± 0.00
**SS-10%**	86.41 ± 0.46	57.71 ± 6.10	30.18 ± 12.26	0.00 ± 0.00	0.00 ± 0.00	0.00 ± 0.00	0.00 ± 0.00

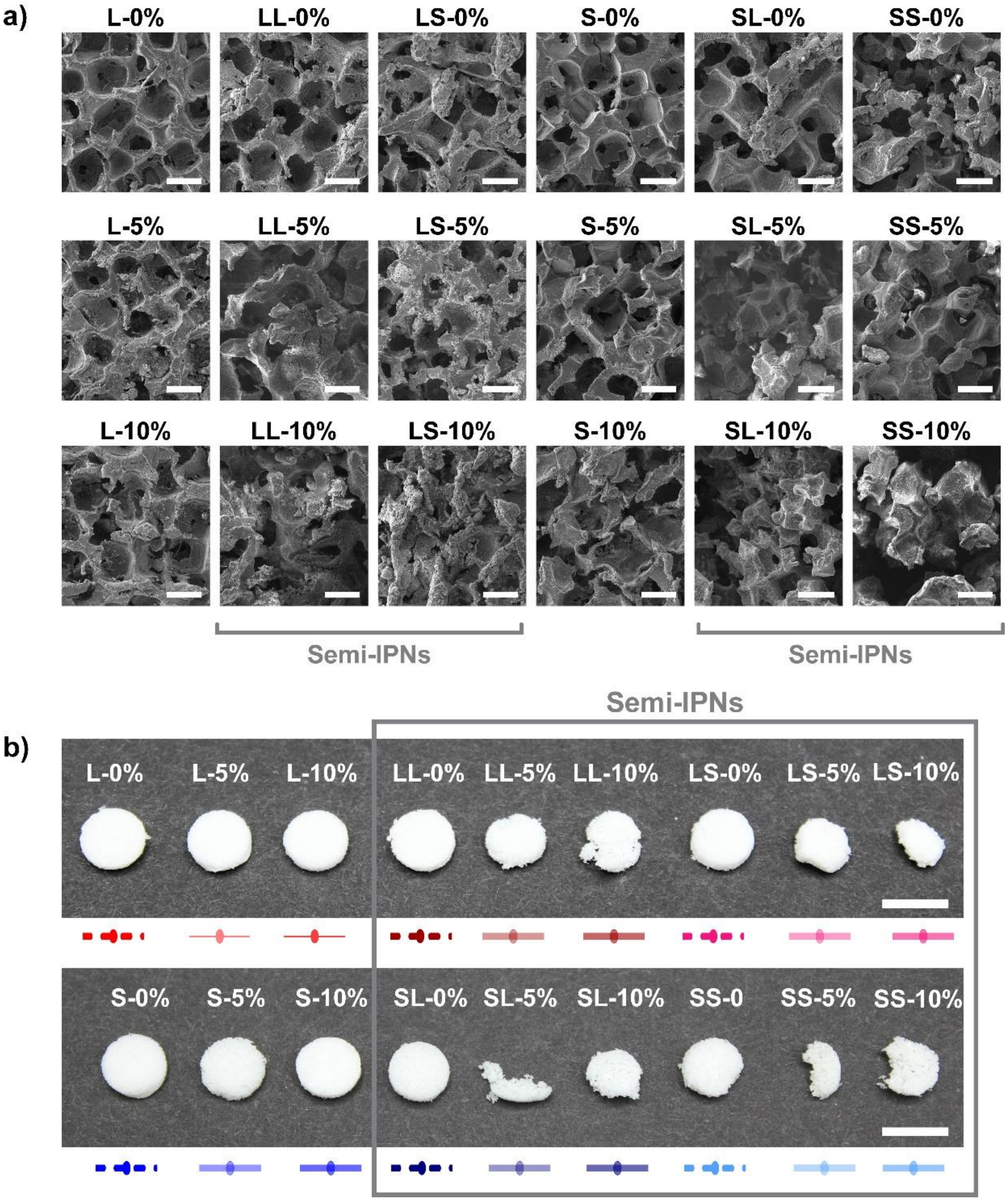

Figure S14. (a) SEM images of accelerated degradation scaffolds after 48 hr (scale bars = 200 μm). **(b)** Photo-series of scaffolds at 48 hr of base-catalyzed degradation study (scale bars = 6 mm).

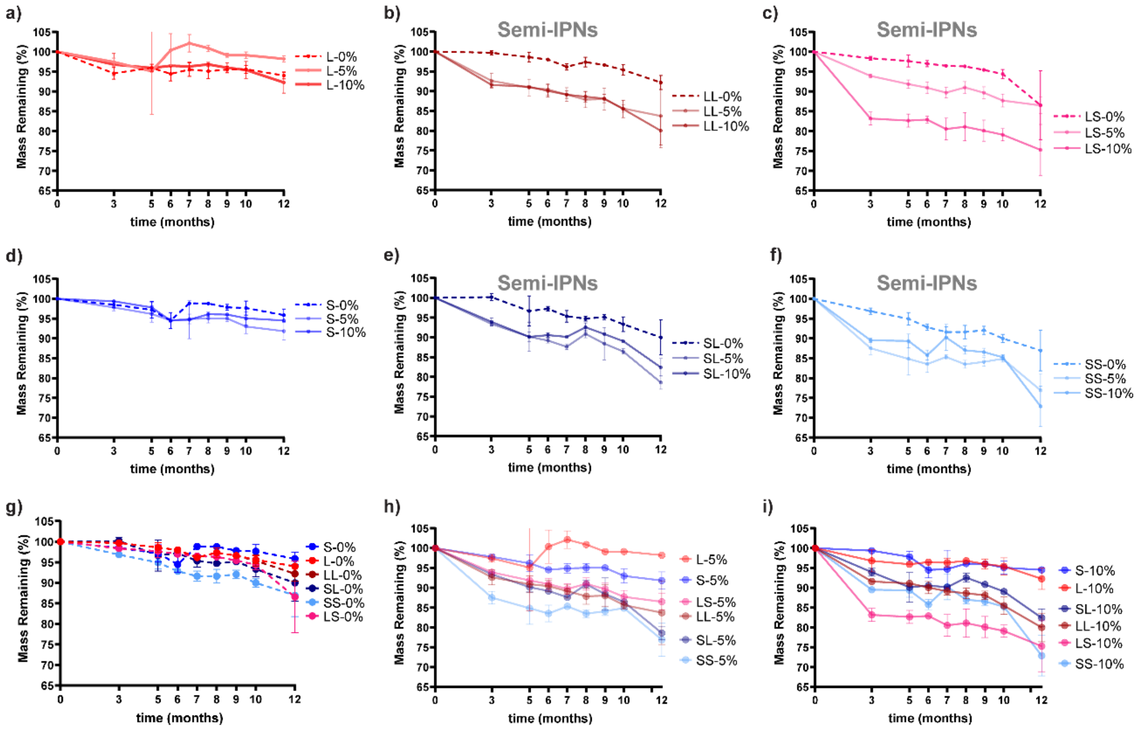

Figure S15. Non-accelerated degradation (1X PBS, 37 °C, 60 rpm) of scaffolds grouped by macromer(s): **(a)** L, **(b)** LL, **(c)** LS, **(d)** S, **(e)** SL, **(f)** SS. Non-accelerated degradation of scaffolds grouped by BG wt%: **(g)** 0% BG, **(h)** 5% BG, and **(i)** 10%.

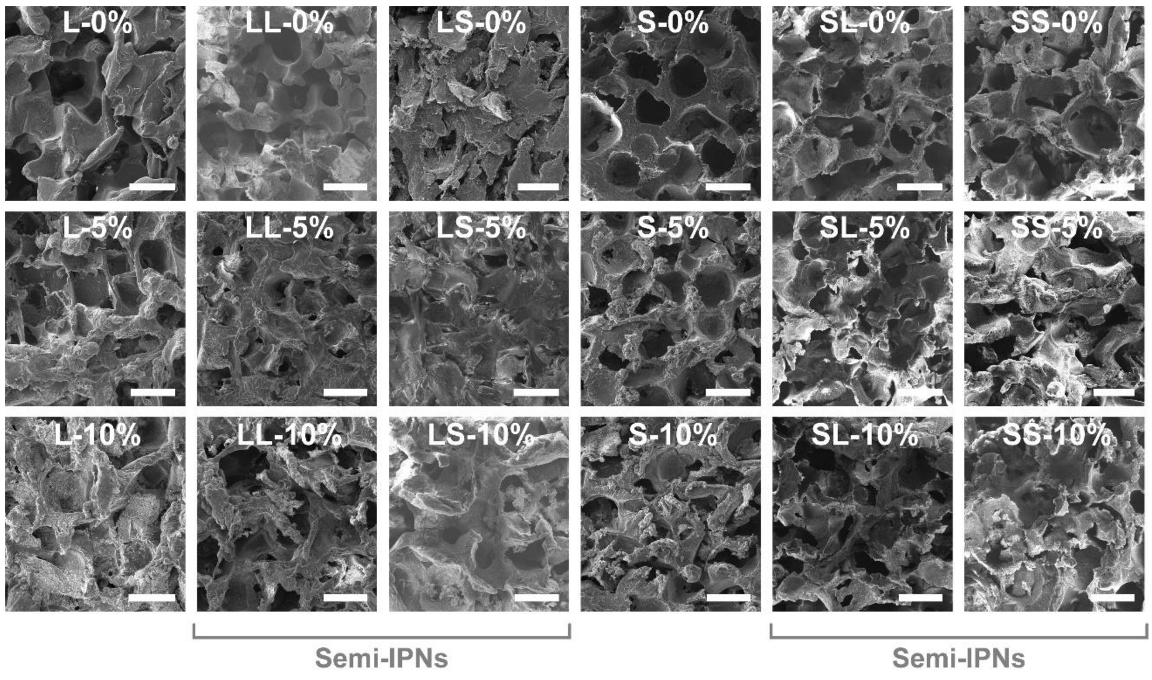

Figure S16. SEM images of non-accelerated degradation scaffolds after 12 months (scale bars = 200 μm).

Table S11. Non-accelerated degradation mass remaining (%) of scaffolds over time.

	3 mo	5 mo	6 mo	7 mo	8 mo	9 mo	10 mo	12 mo
**L-0%**	94.56 ± 1.48	95.95 ± 0.88	94.44 ± 1.79	95.50 ± 1.72	95.11 ± 1.86	95.50 ± 0.79	95.59 ± 1.00	94.00 ± 0.82
**L-5%**	97.43 ± 1.00	95.10 ± 10.90	100.37 ± 4.19	102.12 ± 2.21	100.90 ± 0.78	99.10 ± 0.53	99.13 ± 0.79	98.22 ± 0.78
**L-10%**	96.77 ± 2.81	95.90 ± 0.33	96.47 ± 0.27	96.29 ± 1.11	96.80 ± 0.55	95.98 ± 1.23	95.40 ± 2.15	92.26 ± 2.68
**LL-0%**	99.70 ± 0.52	98.57 ± 1.24	98.00 ± 0.13	96.16 ± 0.84	97.33 ± 1.21	96.61 ± 0.39	95.37 ± 1.34	92.18 ± 1.77
**LL-5%**	92.62 ± 1.87	90.91 ± 2.11	90.43 ± 1.43	89.14 ± 1.73	87.86 ± 2.02	88.00 ± 2.75	85.63 ± 0.96	83.71 ± 8.03
**LL-10%**	91.57 ± 0.65	91.10 ± 0.40	90.07 ± 1.53	89.11 ± 0.93	88.60 ± 1.35	88.12 ± 1.03	85.49 ± 2.18	79.99 ± 3.62
**LS-0%**	98.35 ± 0.46	97.68 ± 1.50	96.99 ± 0.78	96.46 ± 0.06	96.32 ± 0.20	95.44 ± 0.41	94.38 ± 1.14	86.56 ± 8.66
**LS-5%**	93.91 ± 0.46	91.84 ± 1.05	90.92 ± 1.45	89.70 ± 1.33	90.99 ± 1.48	89.67 ± 1.50	87.70 ± 1.63	86.47 ± 2.09
**LS-10%**	83.16 ± 1.65	82.66 ± 1.66	82.89 ± 0.86	80.52 ± 2.74	81.09 ± 3.57	80.12 ± 2.66	79.10 ± 1.53	75.27 ± 6.41
**S-0%**	98.51 ± 0.75	97.18 ± 2.08	94.49 ± 2.01	98.83 ± 0.34	98.78 ± 0.36	97.82 ± 0.76	97.65 ± 1.77	95.87 ± 1.52
**S-5%**	97.80 ± 0.82	96.15 ± 2.07	94.54 ± 0.58	94.90 ± 1.22	95.03 ± 1.13	95.02 ± 0.81	92.99 ± 1.75	91.81 ± 2.18
**S-10%**	99.35 ± 0.26	97.81 ± 1.38	94.49 ± 2.01	94.71 ± 4.78	96.09 ± 0.52	96.00 ± 0.27	95.08 ± 1.65	94.52 ± 0.15
**SL-0%**	100.17 ± 0.88	96.65 ± 3.81	97.22 ± 0.59	95.34 ± 1.47	94.73 ± 0.61	95.14 ± 0.62	93.33 ± 1.86	89.98 ± 4.40
**SL-5%**	93.39 ± 0.47	90.16 ± 1.21	89.18 ± 0.61	87.62 ± 0.74	90.88 ± 0.99	88.41 ± 4.07	86.44 ± 0.71	78.59 ± 1.70
**SL-10%**	93.94 ± 1.00	90.14 ± 3.73	90.57 ± 0.62	90.13 ± 0.33	92.55 ± 1.11	90.84 ± 0.25	89.03 ± 0.39	82.40 ± 2.24
**SS-0%**	96.85 ± 0.80	94.91 ± 1.59	92.83 ± 0.76	91.59 ± 1.28	91.60 ± 1.67	92.03 ± 1.01	89.99 ± 1.04	86.94 ± 5.16
**SS-5%**	87.51 ± 1.60	84.83 ± 4.03	83.53 ± 2.10	85.33 ± 0.63	83.48 ± 0.99	84.08 ± 1.09	84.88 ± 0.81	76.93 ± 4.23
**SS-10%**	89.50 ± 0.61	89.35 ± 1.76	85.76 ± 1.30	90.26 ± 3.28	87.01 ± 1.02	86.55 ± 0.96	85.25 ± 0.71	72.88 ± 5.11

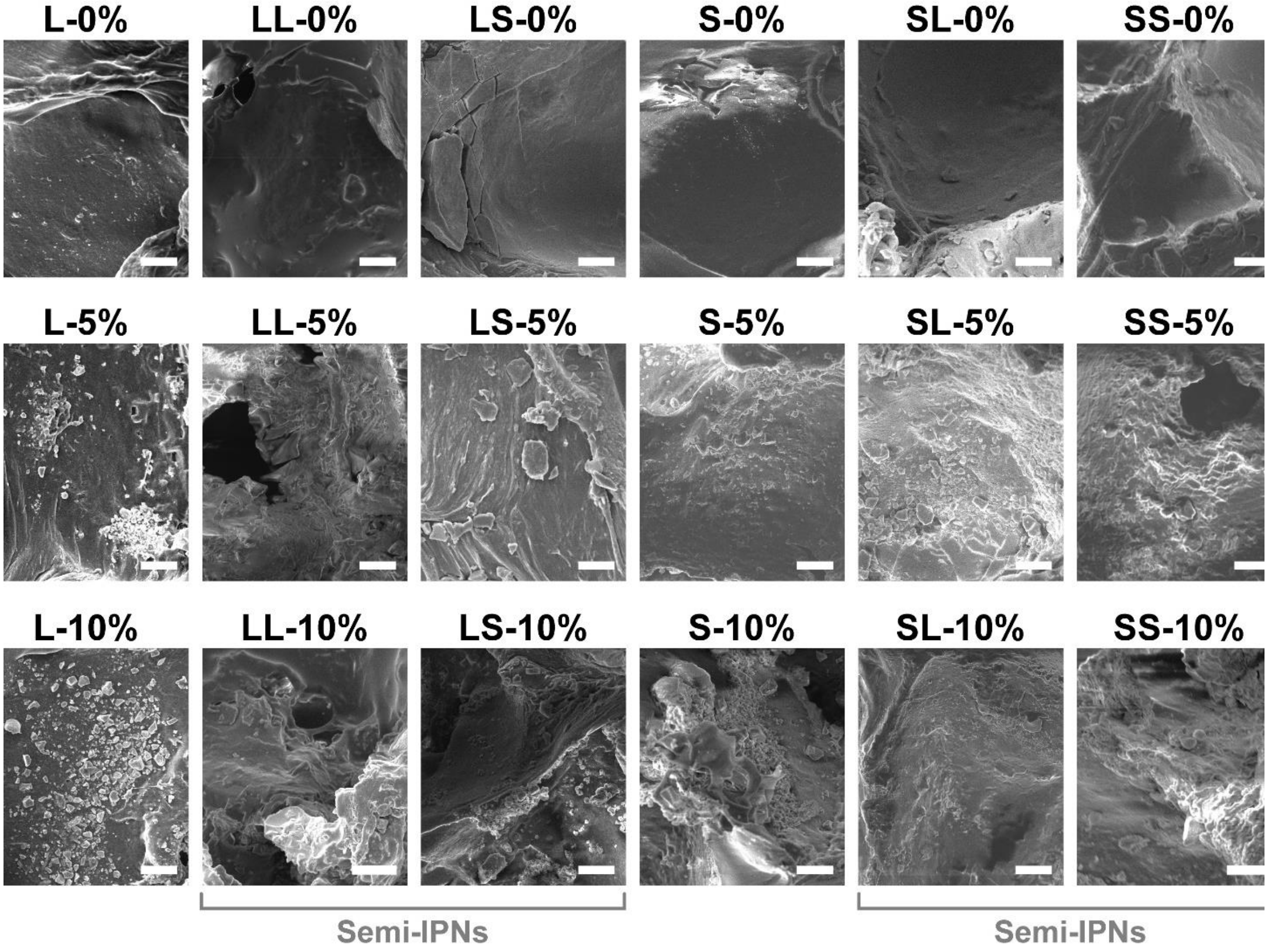

Figure S17. SEM images of scaffolds with no SBF exposure (scale bars = 20 μm).

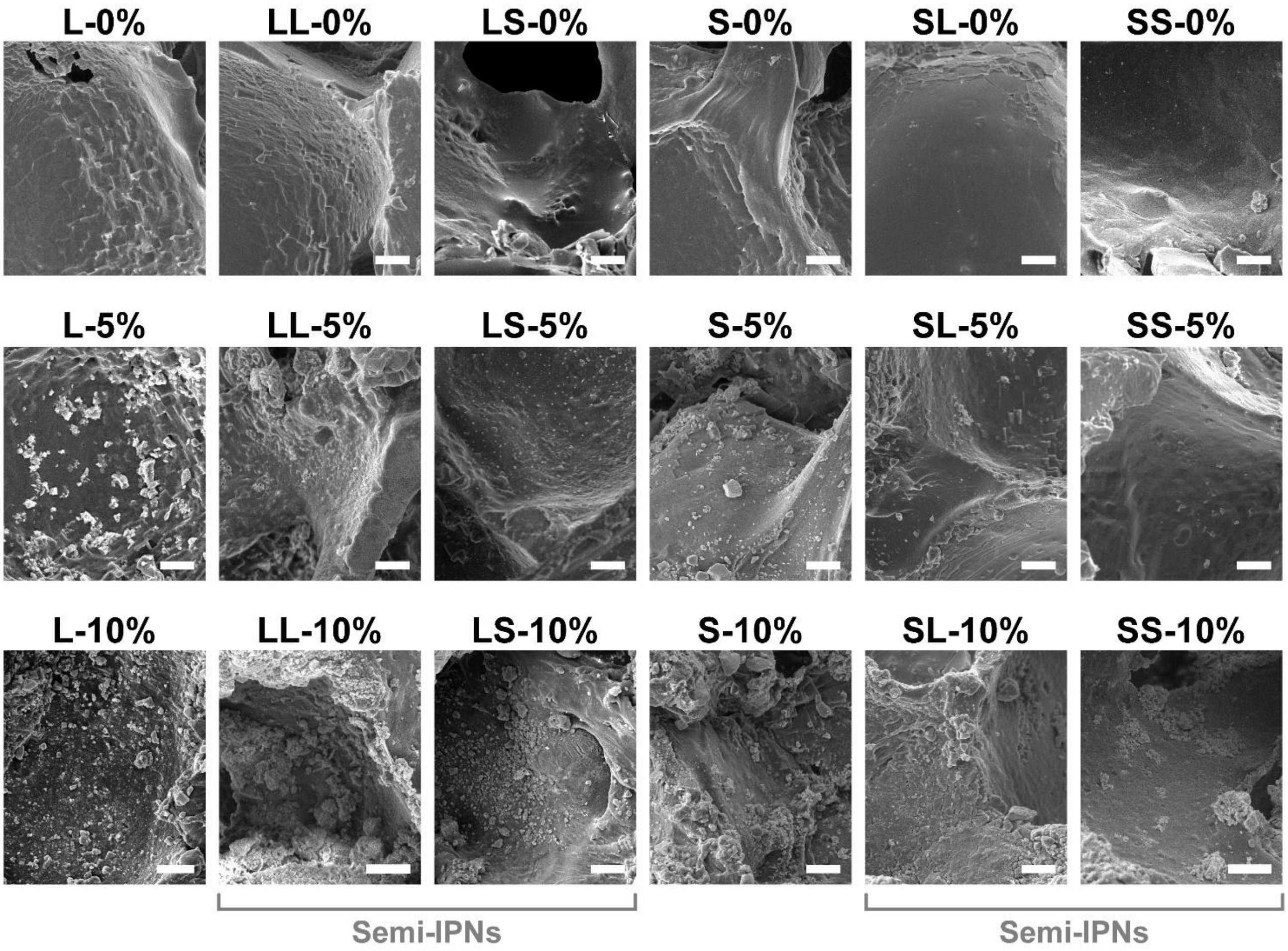

Figure S18. SEM images of scaffolds after 1 day of SBF exposure (scale bars = 20 μm).

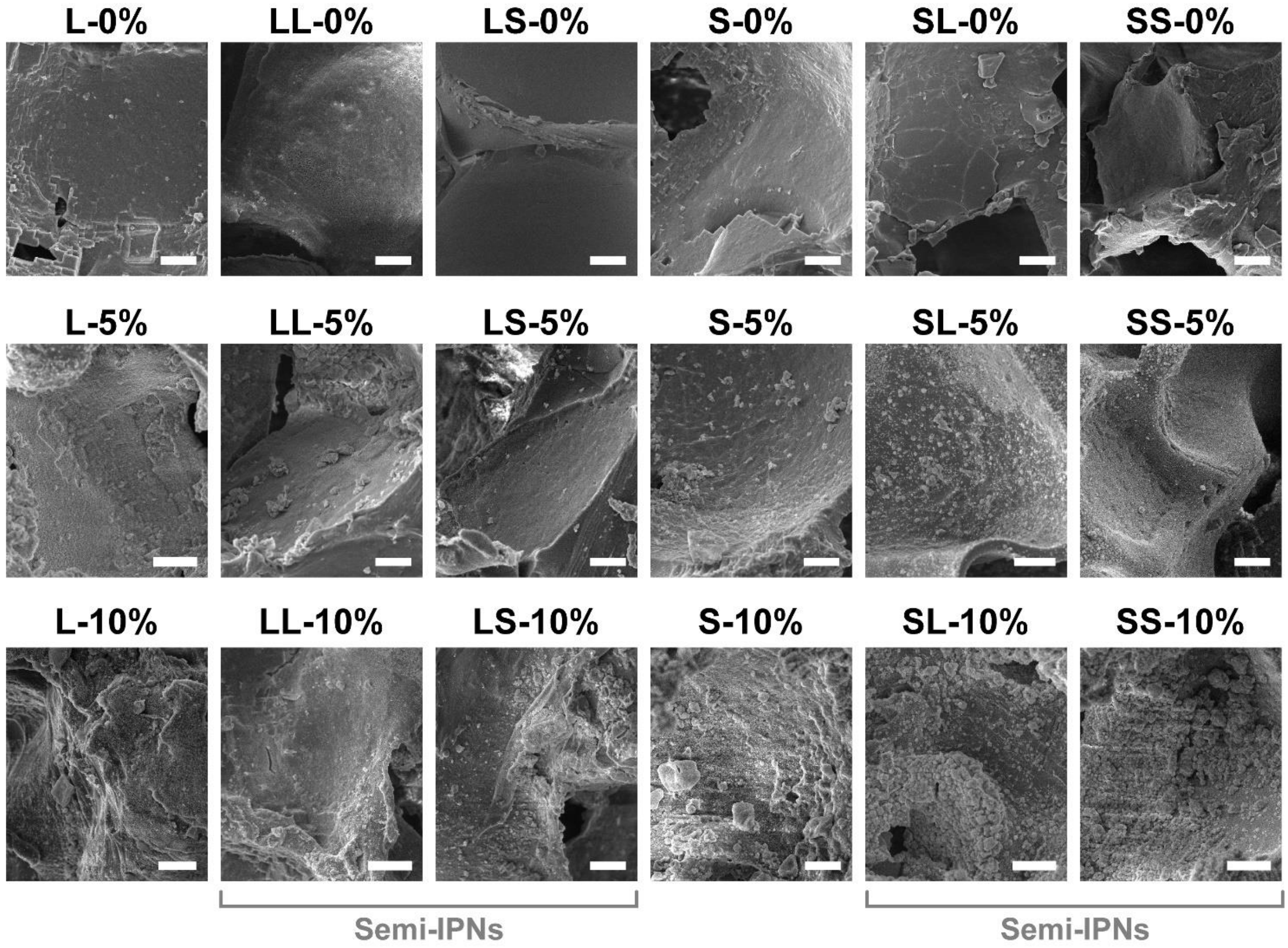

Figure S19. SEM images of scaffolds after 2 weeks of SBF exposure (scale bars = 20 μm).

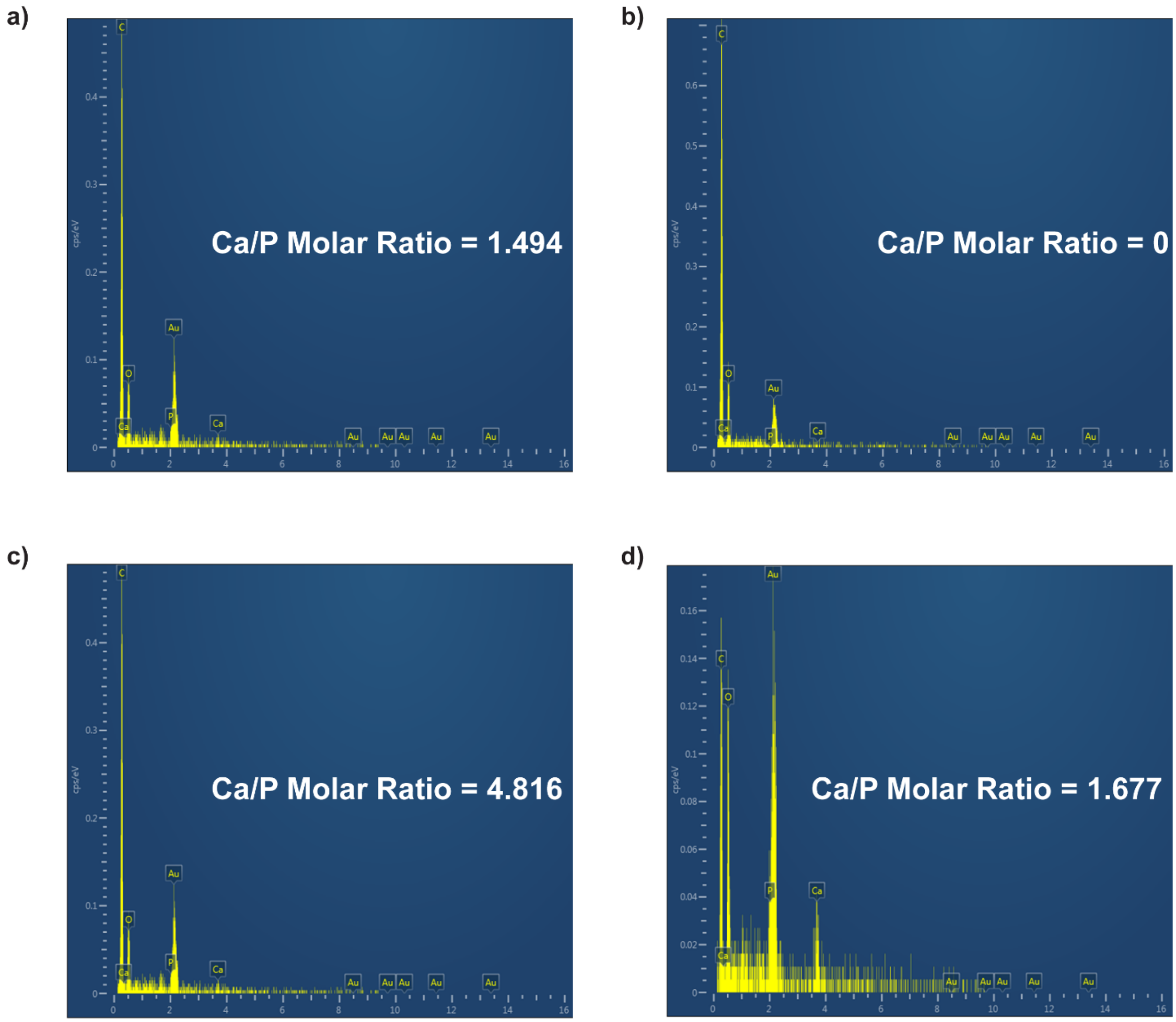

Figure S20. Representative EDS spectra of a **(a)** BG particle on scaffold, **(b)** non-BG-containing scaffold surface after 1 day and 2 weeks in SBF (1X), **(c)** composite scaffold surface [L-5%] after 1 day in SBF (1X), **(d)** composite scaffold surface [L-5%] after 2 weeks in SBF (1X).
